# Leachable-Derived Impurities in Oral Solid Dosage Forms Generated by Solid-State Packaging–Excipient Interactions: A Case Study of a Polyvinyl Chloride Heat Stabilizer Reacting with Povidone

**DOI:** 10.3390/pharmaceutics18091153

**Published:** 2026-09-14

**Authors:** Ewoud Vaneeckhaute, Julie Van Hooste, Pasquinel Weckx, Andrew Teasdale, Ruud Cuyvers, Jonathan Hammond, Daniel Wood, Ward D’Autry, Laura Martin, Eric Breynaert

**Affiliations:** 1 NMRCoRe, NMR/X-Ray Platform for Convergence Research, KU Leuven, Celestijnenlaan 200F-Box 2461, B-3001 Leuven, Belgium; 2 Center for Surface Chemistry and Catalysis—Characterisation and Application Team, KU Leuven, Celestijnenlaan 200F-Box 2461, B-3001 Leuven, Belgium; 3 Nelson Labs, Romeinsestraat 12, B-3001 Leuven, Belgium; 4 Norgine Pharmaceutical Ltd., ARC Uxbridge, Building 01, Sanderson Road, Uxbridge UB8 1DH, UK

**Keywords:** leachables, extractables, oral solid dosage form, blister packaging, polyvinyl chloride, organotin heat stabilizer, povidone, pyrrolidin-2-one, impurity identification, ICH Q3E

## Abstract

**Background/Objectives:** Oral solid dosage (OSD) forms are historically categorized as low-risk vectors for leachables under the assumption that solid-state matrices impose severe kinetic barriers against migrant migration and reactivity. This study investigates an unexpected unknown impurity, detected as part of a routine ICH Q3B testing program to control drug product impurities in a blister-packed tablet. Since the impurity exceeded the ICH Q3B identification threshold applicable for this drug product (1.0% *w*/*w* relative to API or 5 µg total daily intake (TDI), whichever is lower), a forensic investigation into the origins and identity of the impurity was performed. **Methods:** Placebo and packaging line studies were performed to isolate the origin of the impurity. Systematic structure elucidation was carried out using high-resolution mass spectrometry (HRMS), isotopic fine structure analysis, and de novo chemical synthesis. To enable definitive characterization, preparative HPLC was utilized to isolate the compound for high-field 2D-NMR spectroscopy (801 MHz). **Results:** The impurity was proven to be independent of active pharmaceutical ingredient degradation, forming only when tablet excipients were stored in sealed PVC blister packaging. Co-incubation of the PVC heat stabilizer derivative dimethyltin bis(2-ethylhexyl mercaptoacetate) (DMTE) with pyrrolidin-2-one (a povidone excipient degradation product) generated an identical chromatographic and MS/MS spectral profile. NMR spectroscopy unambiguously identified the structure as 2-ethylhexyl 2-((5-oxopyrrolidin-2-yl)thio)acetate. **Conclusions:** This study provides the first direct evidence of an organotin-catalyzed solid-state reaction between a packaging leachable and a tablet excipient under 25 °C/60% RH storage conditions. While this implies that the impurity should not be evaluated under the ICH Q3B guidelines, the findings directly challenge the assumed “low-risk” status of OSD packaging regarding leachables and highlight the necessity for interaction-focused, chemistry-based risk assessments, as outlined in emerging ICH Q3E guidelines.

## 1. Introduction

Evaluation and control of impurities in pharmaceutical products traditionally focus on related substances and impurities arising from the active pharmaceutical ingredient (API) synthesis and/or later drug product manufacturing processes including excipients [1,2,3]. More challenging to assess are impurities originating from materials that come into contact with the drug product, such as extractables and leachables derived from packaging systems, container-closure components, or manufacturing equipment [4,5,6,7]. Consequently, the presence and potential impact of these non-intentionally added substances (NIAS) are less comprehensively addressed in the guidance framework of the International Council for Harmonisation (ICH) for impurity evaluation, summarized in the ICH Q3 guidelines [8,9].

Traditionally, regulatory scrutiny has been ranked by the route of administration. Inhalation (OINDPs) and parenteral drug products (PDPs) are subjected to the most rigorous testing due to the high bioavailability of contaminants and the direct route to systemic circulation [10,11]. In contrast, oral solid dosage (OSD) forms such as tablets and capsules have been historically viewed as “low-risk”, only requiring compliance with food contact regulations up until recently [4]. The assumption driving this risk categorization is that the high viscosity and solid state of the OSD impose kinetic barriers to the diffusion of migrants from the packaging material into the drug product. This paradigm is now shifting. The most recent draft ICH Q3E guideline emphasizes a more holistic, chemistry-based risk assessment that looks beyond simple dosage form categorization [9]. However, as illustrated in this case study, the migration of leachables from packaging into OSD forms should not be underestimated.

The case highlighted here serves as a first report of the generation of a byproduct formed from a complex interaction between the formulation and PVC blister materials commonly used throughout the pharmaceutical industry [12]. The result is the leaching of the byproduct into an OSD form, potentially necessitating a re-evaluation of risk assessments for PVC-blistered tablets.

## 2. Case History

In June 2022, routine stability monitoring of a commercial drug formulation revealed a significant anomaly. An unknown impurity, eluting in reverse-phase UV-HPLC, was quantified at 1.2% (*w*/*w*% relative to the API), breaching the ICH Q3B identification limit. The initial phase of the investigation focused on the potential degradation of the API. HPLC analysis of aged bulk tablets (prior to packing), however, showed no detectable level of the impurity, while a placebo batch, manufactured without API and subsequently blister-packed, exhibited formation of the impurity. This effectively ruled out the API as the source. A targeted packaging line study demonstrated that the impurity was first detected after 1 week of storage in sealed blisters at 25 °C/60% RH. Data obtained in long-term (25 °C/60% RH; 12 months) and accelerated (30 °C/60% RH and 40 °C/75% RH) stability studies did not indicate a temperature or humidity dependence. While the impurity levels initially increased, values at later time points remained within a relatively narrow range, indicating a plateau in impurity formation. Tablets that were blister-packed and immediately de-blistered and stored in amber glass jars, in contrast, did not contain the impurity. Together, these findings indicated that only the tablet excipients and the blister were essential for the formation of the impurity, and that extended exposure increased the impurity concentration.

## 3. Materials and Methods

### 3.1. HPLC-MS

#### 3.1.1. Sample Preparations

##### Tablet Analysis

A total of 96 tablets were transferred into a 50 mL volumetric flask, where 40 mL methanol for LC/MS (LiChrosolv^®^, Merck, Darmstadt, Germany) was added and mixed. The solution with the tablets was sonicated for 5 min, followed by vortexing (VWR 19091S-433UI, VWR, part of Avantor, Leuven, Belgium), before sonicating (VWR 142-6009, VWR, part of Avantor, Leuven, Belgium) for another 5 min. The samples were then centrifuged for 10 min at 2000 rpm (centrifuge: VWR, part of Avantor, Leuven, Belgium), where the supernatant was transferred to an evaporation tube. After concentration to about 2 mL using a Turbovap (Biotage, Uppsala, Sweden), the samples were diluted with methanol to approximately 20 mL. This solution was centrifuged again, and the supernatant was filtered prior to transfer to an evaporation tube for evaporation until dryness. The sample was finally reconstituted with 500 µL of 90/10 (*v*/*v*) ultrapure water/isopropanol (UPW/IPA). UPW was obtained from a MilliQ Advantage A 10 system from Millipore (MilliporeSigma, Burlington, MA, USA) and IPA (AnalaR NORMAPUR) was obtained from VWR (VWR BDH Chemicals, Leuven, Belgium).

##### Reference Standards

**Scheme 1:**
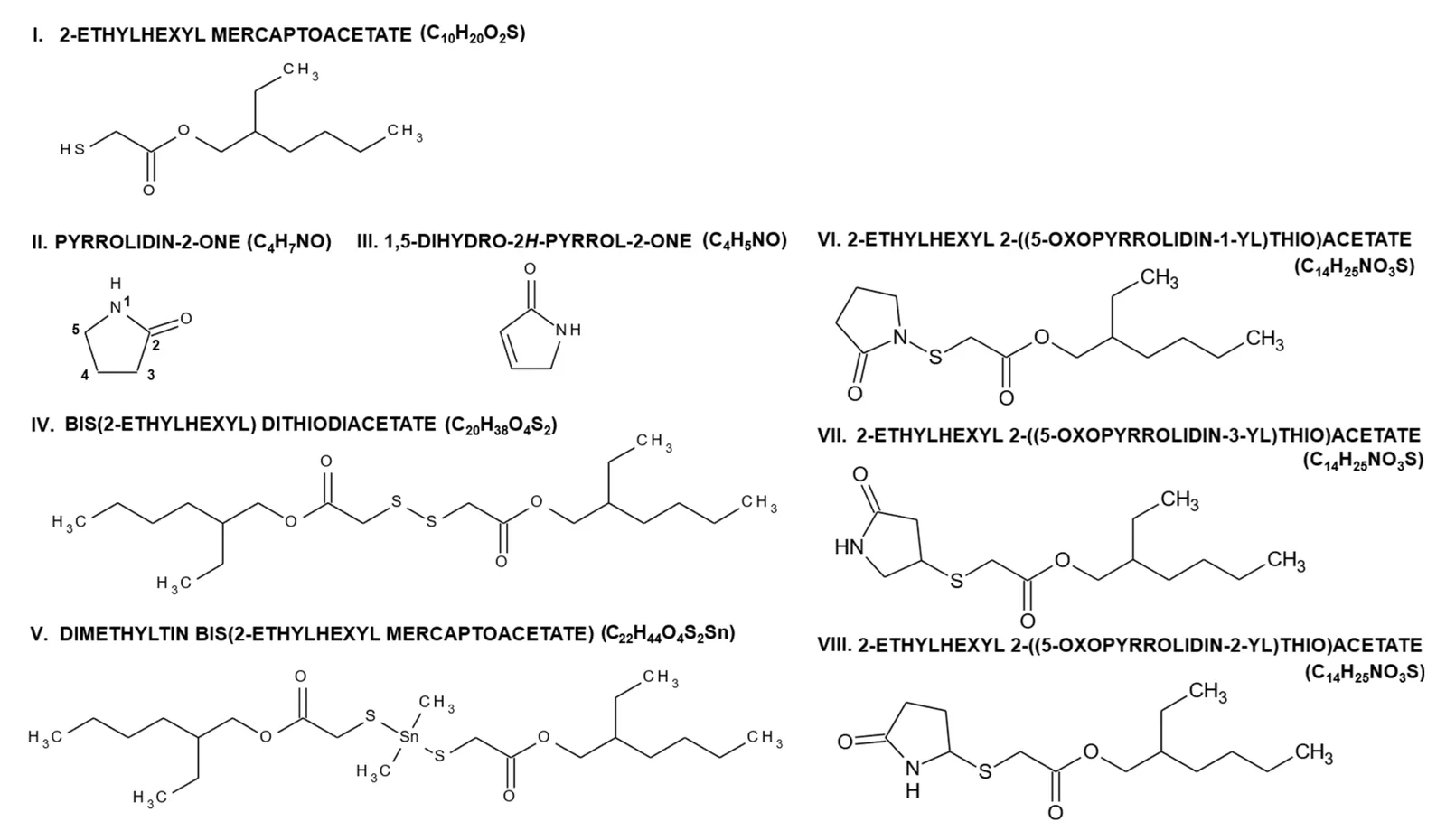
Molecular structures of relevant components identified in the tablet and leachables analysis.

Pyrrolidin-2-one (Scheme 1, **II**) was obtained from TCI Europe (Zwijndrecht, Belgium). Bis(2-ethylhexyl) dithiodiacetate (Scheme 1, **IV**) and dimethyltin bis(2-ethylhexyl mercaptoacetate) (DMTE) (Scheme 1, **V**) were purchased from Angene international Limited (Hong Kong, China). The impurity candidates, 2-ethylhexyl 2-((2-oxopyrrolidin-1-yl)thio)acetate (Scheme 1, **VI**), 2-ethylhexyl 2-((5-oxopyrrolidin-3-yl)thio)acetate (Scheme 1, **VII**) and 2-ethylhexyl 2-((5-oxopyrrolidin-2-yl)thio)acetate (Scheme 1, **VIII**), were custom synthesized and purchased from ChiroBlock GmbH (Wolfen, Germany). Acetone was used as a dissolution solvent and obtained from Merck (Darmstadt, Germany).

##### Internal Standard for Injection (ISI) Solution

An ISI solution was prepared containing 100 ± 10 mg/L caffeine-(trimethyl-^13^C_3_) and 100 ± 10 mg/L terephthalic-2,3,5,6-*d*_4_ acid in methanol. Caffeine-(trimethyl-^13^C_3_) was obtained from Merck Life Science BV (Hoeilaart, Belgium) and was used according to the internal standard in ESI (+) mode. Terephthalic-2,3,5,6-*d*_4_ acid was obtained from J.H. Ritmeester BV (Nuenen, The Netherlands) and was used according to the internal standard in ESI (−) mode.

##### Reference Standard Solutions

Solutions of pyrrolidin-2-one, bis(2-ethylhexyl) dithiodiacetate, DMTE and 2-ethylhexyl 2-((5-oxopyrrolidin-2-yl)thio)acetate measuring 25 µM were prepared in methanol.

##### Preparation of Analytical Samples

Prior to analysis, the reference standard solutions and the tablet sample were spiked with the ISI solution at a ratio of 10 µL ISI/1 mL solution.

#### 3.1.2. UHPLC-MS Instrument and Parameters

All HPLC-MS analyses of samples and standards described in Section 3.1.1 were conducted on a Vanquish™ UHPLC system coupled to a Q-Exactive Orbitrap mass spectrometer (Thermo Fisher Scientific, Waltham, MA, USA). The HPLC-MS system was operated with the following parameters or settings:

- Analytical column: Waters Acquity™ HSS T3^®^-C18 2.1 mm × 100 mm (1.8 µm) (Waters Corporation, Milford, MA, USA).
- Mobile phase A: 0.05% formic acid in ultrapure water (UPW).
- Mobile phase B: 0.05% formic acid in methanol.
- Mobile phase gradient (time|%A|%B|flow rate):
  – 0 min|98% A|2% B|0.25 mL/min;
  – 15.50 min|35% A|65% B|0.25 mL/min;
  – 17.50 min|2% A|98% B|0.25 mL/min;
  – 18.00 min|0% A|100% B|0.25 mL/min;
  – 19.50 min|0% A|100% B|0.50 mL/min;
  – 29.50 min|0% A|100% B|0.50 mL/min;
  – 32.00 min|98% A|2% B|0.25 mL/min;
  – 37.00 min|Stop run.
- Column temperature: 40 °C.
- Injection volume: 5 µL.
- Autosampler temperature: 10 °C.
- MS parameters:
  – Acquisition mode: ElectroSpray Ionization (ESI)|MS1 (+/−), MS1 (−) and MS2;
  – MS1 mass range: 70–700 Da;
  – MS1 resolution: 35,000;
  – MS2 Collision Mode: Higher-Energy Collisional Dissociation (HCD) 35 eV;
  – MS2 resolution: 17,500;
  – Precursor inclusion list for MS2 data: *m/z* −203.1109, +86.0605, +288.1629, +365.2652, +407.2277.

For the preparation of the mobile phases, methanol for LC/MS (LiChrosolv^®^, Merck, Darmstadt, Germany) was used and formic acid was obtained from Fisher Scientific, a part of Thermo Fisher Scientific (Loughborough, UK).

### 3.2. Impurity Isolation by HPLC-UV/MS

To enable NMR for structure elucidation, the target impurity of interest had to be produced in sufficient amounts and isolated. To this end, 3 mL of DMTE (Scheme 1, **V**) and 3 mL pyrrolidin-2-one (Scheme 1, **II**) were mixed with 650 µL of acetone (Merck, Darmstadt, Germany) in a 12 mL glass vial, closed, and incubated for 16 h at 70 °C. This procedure was performed in triplicate to obtain a total volume of 20 mL reactant solution. The reactant solution was distributed in 2 mL autosampler vials with inserts, with each vial containing 125 µL of reactant solution. From each vial (112 in total), 100 µL was injected onto the same instrument as used for the HPLC-MS experiments: a Vanquish™ UHPLC system was coupled to a Q-Exactive Orbitrap mass spectrometer (Thermo Fisher Scientific, Waltham, MA, USA), using an adapted method for impurity isolation. The instrument was operated with the following parameters or settings:

- Analytical column: Supelco Discovery^®^ C8 150 mm × 4.6 mm (5 µm) (Merck, Darmstadt, Germany).
- Mobile phase A: ultrapure water (UPW).
- Mobile phase B: methanol.
- Mobile phase gradient (time|%A|%B|flow rate):
  – 0 min|70% A|30% B|1.00 mL/min;
  – 6.50 min|0% A|100% B|1.00 mL/min;
  – 10.00 min|0% A|100% B|1.00 mL/min;
  – 11.00 min|70% A|30% B|1.00 mL/min;
  – 15.00 min|Stop run.
- Column temperature: 40 °C.
- Injection volume: 100 µL.
- Autosampler temperature: 10 °C.

Using this method, the target impurity of interest eluted at 6.60 min on MS. To collect the peak of the impurity, a valve switch was operated between 6.37 and 6.65 min, where the eluent from the column was redirected to a glass flask (Figures S1–S5). In total, 31 mL of eluent containing the purified target impurity of interest was obtained, as was confirmed by comparing the retention time and the MS2 spectrum of the collected impurity with that of the tablet impurity analysis (Figures S1–S6). Finally, the collected fraction containing the target impurity was evaporated to dryness and distributed for NMR analysis.

### 3.3. NMR Spectroscopy

A measure of 10 mg of the synthesized sample was subjected to comprehensive NMR spectroscopic analysis to identify the regiochemistry of the molecule. For the NMR analysis, aliquots of the purified compound were accurately weighed using a Sartorius CPA225D analytical balance (Sartorius, Göttingen, Germany; precision ±0.01 mg) and dissolved in CDCl_3_ (Merck KGaA, Darmstadt, Germany) to obtain a final concentration of ~140 mg mL^−1^. Samples were transferred to 5 mm NMR tubes (Norell, Morganton, NC, USA). Tetramethylsilane (TMS, Merck KGaA, Darmstadt, Germany) was used as the internal chemical shift reference (*δ*_1H_ = 0.00 ppm). All NMR spectra were recorded on an Avance Neo 800 MHz system (Bruker BioSpin, Billerica, MA, USA) equipped with a 5 mm BBO probe (Bruker BioSpin). The sample temperature was maintained at 298 K using a BCU-II (Bruker BioSpin). The spectrometer was operated in a temperature-controlled environment stabilized at 295 K.

The ^1^H NMR spectra were acquired at 801.25 MHz and the ^13^C NMR spectra were acquired at 201.45 MHz. Standard Bruker pulse sequences were used for the ^1^H, ^13^C, COSY, HSQC, and HMBC experiments. The ^1^H NMR spectra were recorded with the zg30 pulse sequence with 16 scans and 2 dummy scans, a spectral width of 20.13 ppm, a relaxation delay of 1 s, and processed using exponential line broadening of 0.3 Hz. Automatic phase and baseline corrections were applied. The ^13^C NMR spectra were recorded with the zgig30 sequence, with 1024 scans, 4 dummy scans and decoupling during acquisition, a spectral width of 240.91 ppm, a relaxation delay of 4 s, and processed using exponential line broadening of 0.3 Hz. Automatic phase and baseline corrections were applied. The COSY spectrum was acquired using the cosygpppqf pulse sequence with gradient pulses with 4 scans, 16 dummy scans, 2048 complex points and 256 increments. The HSQC spectrum was acquired using a phase-sensitive hsqcedetgpsisp2.3 pulse sequence with a double inept-transfer and multiplicity editing (CH/CH_2_ versus CH_3_) with 4 scans, 32 dummy scans, 2048 complex points and 256 increments. The experiment was optimized for a one-bond heteronuclear coupling constant of ^1^*J*_CH_ = 145 Hz. The HMBC spectrum was acquired using the hmbcgplpndqf pulse sequence with 8 scans, 16 dummy scans, 4096 complex points and 256 increments optimized for long-range couplings of ^n^*J*_CH_ = 10 Hz. For all experiments, non-uniform sampling (NUS) was applied at 25% sampling density in the indirect dimension. The NUS data were reconstructed using the MDD algorithm, yielding a final matrix size of 4k × 4k points after zero-filling. Prior to Fourier transformation, data were apodised using sine-bell-squared window functions with a broadening of 1 Hz in both dimensions and zero-filled to improve digital resolution. Baseline correction and phase correction were applied where appropriate.

## 4. Results and Discussions

### 4.1. Analytical Challenges and the Exclusion of the API

**Figure 1:**
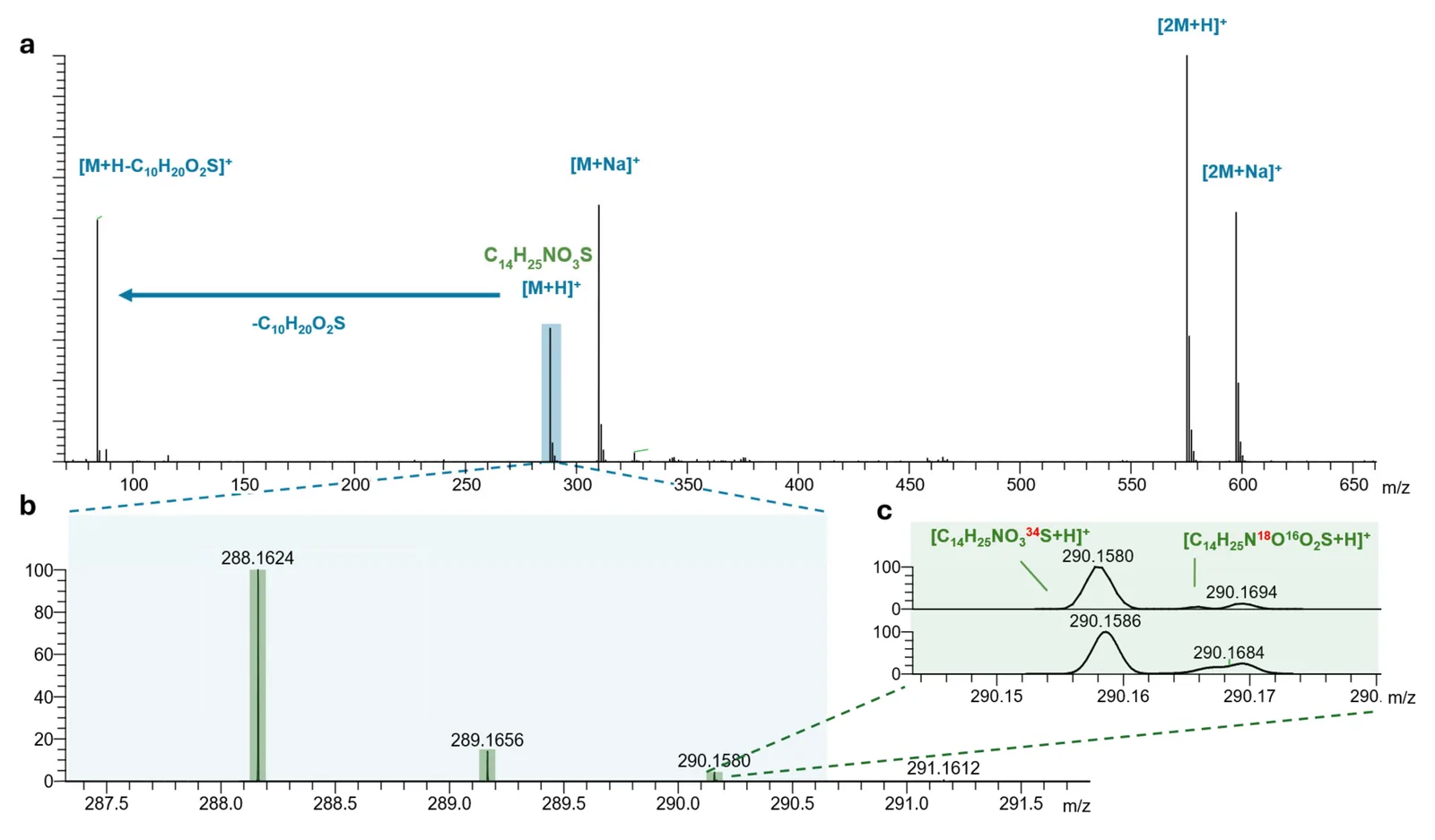
**Structure elucidation of the impurity using high-resolution mass spectroscopy (HRMS)**. **Top**: (**a**) Full-scan MS1 spectrum of the unknown impurity. The blue arrow highlights a neutral loss of C_10_H_20_O_2_S. **Bottom**: (**b**) MS1 spectrum of the impurity, matching the simulated isotope pattern of [C_14_H_25_NO_3_S+H]^+^ (green bars). **Inset**: (**c**) Isotope fine pattern of the A+2 isotope, showing both the experimental (**top**) and simulated (**bottom**) ^34^S isotope- and ^18^O isotope-containing components.

While the level of the impurity exceeded the identification threshold as defined in the ICH Q3B guidelines [13], its low concentration (nanogram levels) prevented direct identification using nuclear magnetic resonance (NMR) spectroscopy. Also, preparative chromatography on the dissolved OSD did not yield a concentration sufficient for NMR identification. Consequently, only high-resolution mass spectrometry (HRMS) was sensitive enough for analysis. Initial HRMS investigations by external laboratories identified a molecular ion with *m/z* +288 (Figure 1a). Based on accurate mass and detailed inspection of the isotope pattern in the MS1 spectrum (Figure 1b,c), indicating the presence of a sulfur atom with high confidence, the elemental composition C_14_H_25_NO_3_S was proposed for the target impurity of interest. A neutral loss in the MS1 spectrum allowed us to identify 2-ethylhexyl mercaptoacetate (C_10_H_20_O_2_S, Scheme 1, **I**) as a substructure, with [C_4_H_6_NO]^+^ as the remaining fragment.

### 4.2. Identification of Precursors and Synthesis of Candidates

Using the formula C_14_H_25_NO_3_S, a search for precursors containing sulfur and nitrogen within the packaging and tablet formulation was attempted.

*Precursor 1 (sulfur source):*

UHPLC-MS analysis of the drug product tablet identified the presence of bis(2-ethylhexyl) dithiodiacetate (C_20_H_38_O_4_S_2_) (Scheme 1, **IV**), eluting in UHPLC-MS at a retention time of 19.84 min with *m/z* +407.2277 (Figure S1). This compound is a dimer of 2-ethylhexyl mercaptoacetate (C_10_H_20_O_2_S) (Scheme 1, **I**). Assuming 2-ethylhexyl mercaptoacetate, also identified as a neutral substructure in the MS analysis, to be a reactant, the nitrogen source was likely to be a C_4_ component.

*Precursor 2 (nitrogen source):*

Alongside bis(2-ethylhexyl) dithiodiacetate (Scheme 1, **IV**), the LCMS analysis of the tablet identified pyrrolidin-2-one (C_4_H_7_NO) (Scheme 1, **II**), eluting at a retention time of 3.38 min with *m/z* +86.0606 (Figure S2).

#### Origins of the Precursors

Polyvinyl chloride (PVC) is the dominant polymer used in pharmaceutical blister packaging owing to its cost-effectiveness, thermoformability, and barrier properties when laminated. However, PVC is inherently unstable to thermal stress [14]. During calendaring (film formation) and thermoforming (blister cavity creation), PVC tends to undergo dehydrochlorination, a “zipper-like” elimination reaction that releases hydrogen chloride (HCl) and forms conjugated polyene sequences, leading to discoloration and mechanical failure [15,16,17]. To mitigate this process, heat stabilizers are ubiquitous in PVC formulations. In the context of high-clarity pharmaceutical films, organotin compounds, specifically tin mercaptides (Scheme 1, **V**), have established themselves as the industry standard due to their high efficiency and ability to maintain transparency [18].

The mechanism of stabilization by these compounds is dual-faceted. Firstly, they act as potent HCl scavengers, neutralizing the acid that would otherwise autocatalyze further degradation. Secondly, and more critically for the context of this investigation, they participate in the substitution of labile chlorine atoms on the PVC backbone (such as allylic chlorides) via a nucleophilic attack, stabilizing the polymer chain [19]. In the case of tin mercaptides, a critical byproduct of this stabilization mechanism, or simply of the thermal oxidation of the stabilizer itself, is the disulfide dimer of the ligand. In the case of stabilizers based on 2-ethylhexyl mercaptoacetate (Scheme 1, **I**), the primary degradation product found in extracts is bis(2-ethylhexyl) dithiodiacetate (Scheme 1, **IV**). The IPA extraction data of the several blister packaging components are indicated further in the Supporting Information (Figure S22) [20].

On the formulation side, povidone (polyvinylpyrrolidone, PVP) and its cross-linked counterpart (polyvinyl polypyrrolidone, PVPP) are functional excipients used as binders and disintegrants. They are synthesized via the free-radical polymerization of *N*-vinylpyrrolidin-2-one (NVP) [21]. While generally considered inert, these polymers carry a specific impurity profile. The monomer *N*-vinylpyrrolidin-2-one can be entrained in PVP during production. Alternatively, it can be formed by the hydrolysis of PVP end groups. Also, thermal degradation can yield *N*-vinylpyrrolidin-2-one [22]. Finally, oxidation products such as peroxides and hydroperoxides are frequently detected in povidone, often originating from the initiation step of polymerization (often using hydrogen peroxide) [23].

### 4.3. Hypothesis for Formation of the Impurity

The impurity is an adduct of the cyclic amide (lactam) of pyrrolidin-2-one (C_4_H_7_NO) (Scheme 1, **II**) and the 2-ethylhexyl mercaptoacetate moiety (C_10_H_19_O_2_S) (Scheme 1, **I**). While pyrrolidin-2-one is chemically stable under neutral conditions, the nitrogen (Scheme 1, **II**, 1st position) and the position alpha to the carbonyl (Scheme 1, **II**, 3rd position) are sites of potential reactivity. The interaction between these excipient-derived impurities and packaging leachables is little understood, with little evidence provided in the literature. Yet, incubating an acetone-based mixture of bis(2-ethylhexyl) dithiodiacetate (Scheme 1, **IV**) and pyrrolidin-2-one (Scheme 1, **II**) standards at 50 °C (see Section 3.1.1, reaction mix 1), the target impurity was formed in trace amounts. While direct synthesis attempts failed to generate the impurity, replacing bis(2-ethylhexyl) dithiodiacetate (Scheme 1, **IV**) with dimethyltin bis(2-ethylhexyl mercaptoacetate) (DMTE) (Scheme 1, **V**), the heat stabilizer used in the PVC blister foil, increased the generation of the impurity by one order of magnitude (Figure S21), which points towards the importance of Sn playing a potential role as catalyst in the reaction.

Guided by the molecular formula of C_14_H_25_NO_3_S, structure elucidation was initiated. Two primary regiochemical scenarios, *N*-substitution and substitution on the 4th position of the pyrrolidin-2-one ring, were explored through de novo synthesis.

#### 4.3.1. The N-Substituted Hypothesis

The first proposed structure involved a connection between the nitrogen atom and the sulfur-containing moiety forming 2-ethylhexyl 2-((5-oxopyrrolidin-1-yl)thio)acetate (Scheme 1, **VI**). This hypothesis was supported by reports in the literature describing the reaction of an alkyl sulfide with pyrrolidin-2-one in the presence of a chlorinating agent [24]. Given that hydrogen chloride can be generated during the degradation of PVC, it is plausible that a similar reaction pathway occurred between these two extractables under the conditions present in the polymer matrix. The UPLC-MS analysis of a 2-ethylhexyl 2-((5-oxopyrrolidin-1-yl)thio)acetate standard (Scheme 1, **VI**), obtained from ChiroBlock GmbH, however, revealed no exact retention time (RT) match or MS/MS spectral match with the unknown impurity (Figure S3).

#### 4.3.2. The C-Substituted Hypothesis

An alternative hypothesis involved the substitution at the forth position of pyrrolidin-2-one. Based on the detection of a compound with the molecular formula C_4_H_5_NO, an alternative structure was suggested. This compound, with two hydrogens less than pyrrolidin-2-one, could be 1,5-dihydro-2*H*-pyrrol-2-one (Scheme 1, **III**). If it is indeed present, it could undergo a thio-Michael addition with 2-ethylhexyl mercaptoacetate (Scheme 1, **I**). In such a mechanism, the nucleophilic sulfur atom attacks the electrophilic double bond of the 1,5-dihydro-2*H*-pyrrol-2-one one (Scheme 1, **III**), to form 2-ethylhexyl 2-((5-oxopyrrolidin-3-yl)thio)acetate (Scheme 1, **VII**). The compound was obtained from ChiroBlock GmbH, but again, neither the RT in LC nor the MS/MS spectrum of this product matched the unknown impurity (Figure S3) [25].

Following the unsuccessful attempts to identify the impurity, an organotin-catalyzed reaction was chosen as the reason for the generation of the impurity. Consequently, a reaction with the Sn-containing DMTE (Scheme 1, **V**) and pyrrolidin-2-one (Scheme 1, **II**) was performed at 50 °C (see Section 3.1.1, reaction mix 2). This reaction increased the formation of the impurity, producing the exact *m/z* +288.162 parent and identical MS/MS spectrum exhibited by the unknown impurity in the tablet. The compound also co-eluted together with the impurity in the tablet extract at 18.75 min, as can be seen in Figure S4.

### 4.4. NMR Identification of the Synthesized Impurity

**Figure 2:**
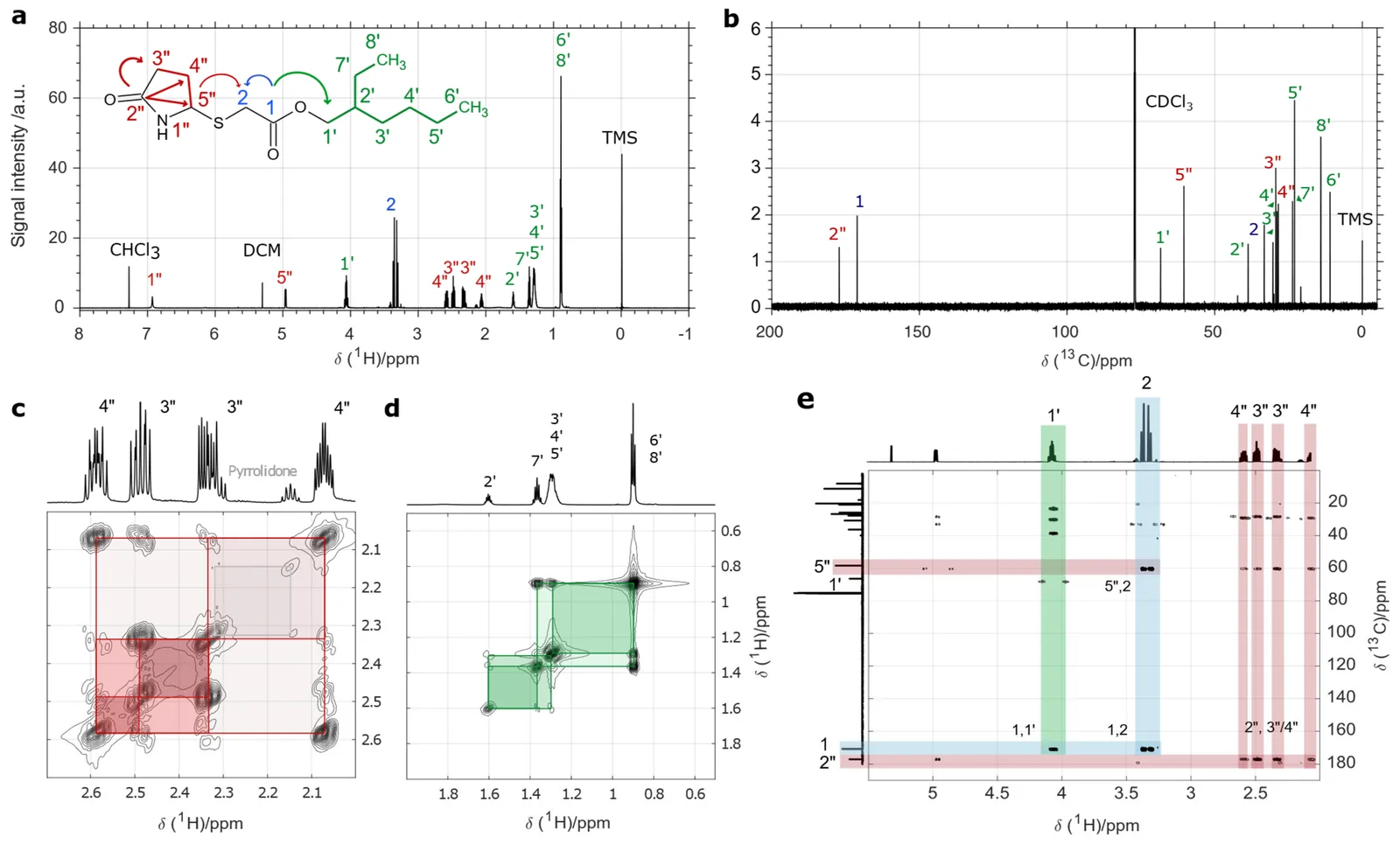
**Structure elucidation of the impurity 2-ethylhexyl 2-((5-oxopyrrolidin-2-yl)thio)acetate using 1D and 2D NMR**. **Top**: (**a**) ^1^H NMR of the isolated impurity dissolved in chloroform-d measured at 298 K and 801 MHz. (**b**) ^13^C NMR of the isolated impurity dissolved in chloroform-d measured at 298 K and 201 MHz). ^1^H and ^13^C chemical shifts can be found in Table S1. **Bottom**: (**c**) ^1^H–^1^H COSY of the pyrrolidine ring. (**d**) ^1^H–^1^H COSY of the branched alkyl chain. Correlations are highlighted with bold bonds onto the Lewis structure. The full ^1^H–^1^H COSY plot is presented in Figure S13. (**e**) Multiple-bond ^1^H–^13^C HMQC of the isolated impurity. Multiple-bond ^1^H–^13^C HMBC correlations are highlighted with arrows onto the Lewis structure. A larger representation of the HMBC spectrum is given in Figure S20 for clarity.

For final structural identification, 10 mg of the purified impurity (cf. Section 3.2) was dissolved in 500 µL of CDCl_3_ with tetramethylsilane (TMS) as reference (*δ*_1H_ = 0 ppm), reaching a final concentration of 140 mM. The planar structure was elucidated using a combination of ^1^H NMR (801 MHz), ^13^C NMR (201 MHz), ^1^H–^1^H COSY, ^1^H–^13^C HSQC, and ^1^H–^13^C HMBC, with the experiments measured at 298 K. The ^1^H and ^13^C peak assignments are reported in Table S1 with detailed representations of the aforementioned 1D and 2D ^1^H and ^13^C NMR, as shown in Figures S7–S20. An overview of the most important NMR results is summarized in Figure 2, comprising 1D ^1^H-NMR (Figure 2a) and ^13^C-NMR (Figure 2b) as well as the 2D ^1^H–^1^H COSY (Figure 2c,d) and ^1^H–^3^C HMBC (Figure 2e) experiments.

An analysis of the COSY spectrum in Figure S15 revealed two independent spin systems. The first spin system, shown in red on the Lewis structure of Figure 2a, corresponds to the pyrrolidone ring, comprising 5 protons on 4 carbons, of which the lactam carbonyl is in the 2”-position. Both H-3”/H-4” are neighboring diastereotopic methylene protons with mutual corresponding cross-peaks, as shown in detail in Figure 2c. This debunks any other substitution than on the 5”-position. The coupling of both H-4” protons to H-5” can be seen in in the full COSY spectrum in Figure S15. The downfield chemical shift (*δ*) of H-5” at 4.96 ppm is explained by both nitrogen and sulfur being adjacent.

The second spin system shown in green on the Lewis structure of Figure 2a consists of a branched alkyl chain extending from H-1′ to the terminal methyl protons H-6′ and H-8′, as shown in the detailed COSY spectrum in Figure 2d. Branching at H-2′ is indicated by the cross-peak between H-2′ and the methyl group H-7′. The HSQC spectrum (Figure S16 and zoomed-in images in Figures S17 and S18) enabled the assignment of all protonated carbons (listed in Table S1), while two non-protonated carbonyl carbons of the pyrrolidinone ring (C2”) and the thioacetate linker (C1) were identified by longer-range HMBC correlations (Figure 2e and Figure S19). Also, their absence in the HSQC spectrum and their characteristic ^13^C chemical shifts at *δ*^13^_C_~170–178 ppm immediately pointed towards carbonyl functionalities. The presence of the lactam carbonyl (C2”, *δ*^13^_C_:177.16 ppm) and its HMBC correlations to H-3” and H-4” again confirm the preservation of the five-membered ring (see Figure 2e).

The connectivity between both spin systems (pyrrolidinone ring in red and the branched alkyl chain in green) was verified by the HMBC experiment show in Figure 2e (enlarged version in Figure S20). The C1 carbonyl carbon in blue is central in the connection due to correlation both to the methylene protons (H-1′, green in Figure 2e) and the thio-methylene protons (H-2, blue in Figure 2e). The downfield chemical shift of C-1′ (*δ*^13^_C_: 68.32 ppm) and H-1′ (*δ*_1H_: 4.07 ppm) supports the presence of an ester linkage between the pyrrolidinone ring and the alkyl chain. The HMBC correlation between and the methine carbon (C-5”, *δ*^13^_C_: 60.42 ppm) and the thio-methylene protons (H-2, *δ*^1^_H_: 3.34 ppm) is the primary evidence for the substitution at the 5”-position and is shown in red in Figure 2e. Together, these data unambiguously identified the planar structure of the impurity as 2-ethylhexyl 2-((5-oxopyrrolidin-2-yl)thio)acetate (Scheme 1, **VIII**), a substituted pyrrolidinone bearing thioether-linked acetate ester with a branched alkyl group.

## 5. Conclusions

This investigation identified a previously undocumented chemical interaction between blister packaging materials and the solid-state matrix of a commercial OSD formulation. The resulting impurity, 2-ethylhexyl 2-((5-oxopyrrolidin-2-yl)thio)acetate, was shown to arise independently of API degradation through reaction of the Sn-containing PVC heat stabilizer derivative DMTE with pyrrolidin-2-one, formed from povidone degradation. The substantially higher impurity yield observed in the presence of DMTE compared with the corresponding non-tin analogue further suggests a potential catalytic contribution of the organotin (Sn) center to the reaction pathway.

Even though, at present, the potential regulatory significance of this impurity remains unclear, the occurrence of this impurity under routine storage conditions highlights that OSD forms are not inherently protected from packaging-related chemical risk. This case study therefore highlights a scenario for which there is currently limited regulatory guidance. While impurities arising from materials extracted or leached from the container closure system are considered within the extractables and leachables risk assessment framework covered by ICH Q3E, oral solid dosage forms are generally considered lower-risk products. This is particularly critical to cases where a secondary impurity is formed through interaction between a leachable and a formulation component.

Draft ICH Q3E guidance provides a framework for the assessment of extractables and leachables, in which oral solid dosage forms are generally considered lower-risk products. The present case study, however, demonstrates that packaging-related interactions can nevertheless occur in oral solid dosage forms and may result in the formation of impurities detected during routine stability studies. The findings of this specific case study therefore directly challenge the long-standing assumption that OSD forms possess a solid-state kinetic barrier sufficient to prevent significant leaching or subsequent reaction. The levels reached by the initially unknown impurity demonstrate that this assumed kinetic barrier does not necessarily represent a reliable threshold for investigation, particularly where reactive or catalytically active residues may be present within packaging systems. Oral solid dosage forms should therefore not automatically be assumed to present negligible extractables and leachables risk. Instead, an appropriate baseline assessment of potential packaging-formulation interactions should be performed before assigning a product-specific risk classification.

From an analytical perspective, these findings also emphasize the need for appropriate method selection. A dedicated, sufficiently sensitive and selective analytical method for drug substance and impurity detection is essential to ensure emerging or low-level leachable-derived species are reliably identified. While a less discriminative detection wavelength can serve routine stability monitoring of already registered products, where the objective is to screen for the presence of peaks from unknown substances, it may not provide adequate specificity during the development or investigation phases. In these instances, a multi-tiered approach combining LC-UVVIS, LC-MS and NMR spectroscopy should be considered.

Especially for new products, a more rigorous approach is required. Full extractables and leachables studies, supported by highly discriminative analytical methodologies, should be performed prior to registration to characterize potential interactions comprehensively and minimize the risk of unidentified impurity formation post-approval. From a broader industry perspective, this work supports a necessary shift toward interaction-focused, science-based risk assessment strategies for OSD products, aligned with the intent of ICH Q3E. Greater emphasis on packaging composition, excipient impurity profiles, and their combined chemical reactivity will be essential to ensure patient safety and regulatory compliance. Collectively, these findings establish an important precedent for increased chemical scrutiny of solid dosage forms packaged in polymer-based systems.

## Data Availability

The original contributions presented in this study are included in the article/Supplementary Materials. Further inquiries can be directed to the corresponding author.

## Supplementary Materials

The following supporting information can be downloaded at https://www.mdpi.com/article/10.3390/pharmaceutics18091153/s1, Figure S1: CHROMATOGRAMS: Top: Extracted Ion Chromatogram (EIC) of *m/z* +407.229 from the analysis of a reference standard of bis(2-ethylhexyl) dithiodiacetate (black); Bottom: EIC of *m/z* +407.2277 from the analysis of the drug product tablet (blue). MS SPECTRA: Top: MS/MS spectrum of precursor *m/z* +407.2 at 19.84 min from the analysis of a reference standard of bis(2-ethylhexyl) dithiodiacetate with relevant annotations of neutral losses (blue) and fragment ion formulas (green); Bottom: MS/MS spectrum of precursor *m/z* +407.2 at 19.84 min from the analysis of the drug product tablet with relevant annotations of neutral losses (blue) and fragment ion formulas (green). Figure S2: CHROMATOGRAMS: Top: Extracted Ion Chromatogram (EIC) of *m/z* +86.060 from the analysis of a reference standard of pyrrolidin-2-one (black); Bottom: EIC of *m/z* +86.060 from the analysis of the drug product tablet (blue). MS SPECTRA: Top: MS/MS spectrum of precursor *m/z* +86.1 at 3.43 min from the analysis of a reference standard of pyrrolidin-2-one with relevant annotations of fragment ion formulas (green); Bottom: MS/MS spectrum of precursor *m/z* +86.1 at 3.38 min from the analysis of the drug product tablet with relevant annotations of fragment ion formulas (green). Figure S3: CHROMATOGRAMS: Top: Extracted Ion Chromatogram (EIC) of *m/z* +288.162 from the analysis of the drug product tablet (black); Middle: EIC of *m/z* +288.162 from the analysis of the synthesized reference standard of 2-ethylhexyl 2-((5-oxopyrrolidin-1-yl)thio)acetate (blue); Bottom: EIC of *m/z* +288.162 from the analysis of the synthesized reference standard of 2-ethylhexyl 2-((5-oxopyrrolidin-3-yl)thio)acetate (green). MS SPECTRA: Top: MS/MS spectrum of precursor *m/z* +288.2 at 18.80 min from the analysis of the drug product tablet with relevant annotations of fragment ion formulas (green); Middle: MS/MS spectrum of precursor *m/z* +288.2 at 18.85 min from the analysis of the synthesized reference standard of 2-ethylhexyl 2-((5-oxopyrrolidin-1-yl)thio)acetate with relevant annotations of fragment ion formulas (green); Bottom: MS/MS spectrum of precursor *m/z* +288.2 at 18.72 min from the analysis of the synthesized reference standard of 2-ethylhexyl 2-((5-oxopyrrolidin-3-yl)thio)acetate with relevant annotations of fragment ion formulas (green). Figure S4: CHROMATOGRAMS: Top: Extracted Ion Chromatogram (EIC) of *m/z* +288.162 from the analysis of reaction mix 2 (pyrrolidin-2-one and DMTE) (black); Bottom: EIC of *m/z* +288.162 from the analysis of the drug product tablet (blue). MS SPECTRA: Top: MS/MS spectrum of precursor *m/z* +288.2 at 18.80 min from the analysis of reaction mix 2 (pyrrolidin-2-one and DMTE) with relevant annotations of fragment ion formulas (green); Bottom: MS/MS spectrum of precursor *m/z* +288.2 at 18.80 min from the analysis of the drug product tablet. Figure S5: Extracted Ion Chromatogram (EIC) chromatogram of *m/z* +288.162 from the analysis of the synthesized impurity using the HPLC-UV/MS method adapted for the isolation of the target impurity of interest, as described in §3.2. The valve switch timing is indicated in blue, where the LC eluent is collected for impurity isolation. The timing is based on the MS retention time window of the target impurity of interest, taking into account a 0.10 min delay between the valve and MS detection. Figure S6: CHROMATOGRAMS: Top: Extracted Ion Chromatogram (EIC) of *m/z* +288.162 from the analysis of the collected fraction after preparative HPLC (black); Bottom: EIC of *m/z* +288.162 from the analysis of the drug product tablet (blue). MS SPECTRA: Top: MS/MS spectrum of precursor *m/z* +288.2 at 18.79 min from the collected fraction after preparative HPLC; Bottom: MS/MS spectrum of precursor *m/z* +288.2 at 18.80 min from the analysis of the drug product tablet. Table S1: Overview of the ^1^H and ^13^C NMR signals observed for 140 mM 2-ethylhexyl 2-((5-oxopyrrolidin-2-yl)thio)acetate referenced towards tetramethylsilane (TMS) in deuterated chloroform (CDCl_3_. Figure S7: Detail of ^1^H NMR spectrum from −0.5 to 2 ppm of the purified impurity (801 MHz, 298K, CDCl_3_). Figure S8: Detail of ^1^H NMR spectrum from 2 to 4 ppm of the purified impurity (801 MHz, 298K, CDCl_3_). Figure S9: Detail of ^1^H NMR spectrum from 4 to 6 ppm of the purified impurity (801 MHz, 298K, CDCl_3_). Dichloromethane (DCM) was found as an impurity common in CDCl_3_. Figure S10: Detail of ^1^H NMR spectrum from 6 to 8 ppm of the purified impurity (801 MHz, 298K, CDCl_3_). Trace protonated chloroform is shown here. Figure S11: Detail of ^13^C NMR spectrum from −5 to 50 ppm of the purified impurity (201 MHz, 298K, CDCl_3_). Figure S12: Detail of ^13^C NMR spectrum from 50 to 100 ppm of the purified impurity (201 MHz, 298K, CDCl_3_). Figure S13: Detail of ^13^C NMR spectrum from 100 to 150 ppm of the purified impurity (201 MHz, 298K, CDCl_3_. Figure S14: Detail of ^13^C NMR spectrum from 150 to 200 ppm of the purified impurity (201 MHz, 298K, CDCl_3_). Figure S15: ^1^H–^1^H COSY spectrum of the purified impurity (801 MHz, 298K, CDCl_3_) with 2048 points in F2 and 256 points in F1 with 25% NUS. Figure S16: ^1^H–^13^C HSQC spectrum of the purified impurity (801 MHz and 201 MHz, 298K, CDCl_3_) with 2048 points in F2 and 256 points in F1 with 25% NUS. Blue correlations are CH/CH3 multiplicity-edited. Black correlations are CH2 multiplicity-edited. Figure S17: Detail of ^1^H–^13^C HSQC spectrum from 0.5 to 2 ppm in F2 and 5 to 45 ppm in F1 of the purified impurity (801 MHz and 201 MHz, 298K, CDCl_3_). Blue correlations are CH/CH3 multiplicity-edited. Black correlations are CH2 multiplicity-edited. Figure S18: Detail of ^1^H–^13^C HSQC spectrum from 1.8 to 2.8 ppm in F2 and 27 to 31 ppm in F1 of the purified impurity (801 MHz and 201 MHz, 298K, CDCl_3_). Black correlations are CH2 multiplicity-edited. Figure S19: ^1^H–^13^C HMBC spectrum of the purified impurity (801 MHz and 201 MHz, 298K, CDCl_3_) with 4096 points in F2 and 256 points in F1 with 25% NUS. A detailed plot is shown in Figure S20. Figure S20: Detail ^1^H–^13^C HMBC spectrum from 2 ppm to 5.5 ppm of the purified impurity (801 MHz and 201 MHz, 298K, CDCl_3_) with 4096 points in F2 and 256 points in F1 with 25% NUS. Figure S21: Top: Extracted Ion Chromatogram (EIC) of *m/z* +288.161 from the analysis of reaction mix 1 (equimolar 2500 µM amounts of pyrrolidine-2-one & bis(2-ethylhexyl) dithiodiacetate) (black). Bottom: EIC of *m/z* +288.161 from the analysis of reaction mix 2 (equimolar 2500 µM amounts of pyrrolidine-2-one & DMTE) (blue). Figure S22: LC/MS data of several blister packaging components after closed vessel incubation extraction (submersion) in isopropanol (IPA). Extraction Ion Chromatograms (EIC) of *m/z* −203.111, the diagnostic ion for DMTE, from top to bottom: The IPA extraction blank; The PVC base foil; The printed aluminum top foil; The non-printed blister (PVC base + aluminum top foil); The printed blister (PVC base + aluminum top foil); An authentic reference standard of DMTE (the small retention time difference can be explained by the fact that this analysis has not run in the same sequence).

## Abbreviations

API	Active Pharmaceutical Ingredient
COSY	Correlation Spectroscopy
DCM	Dichloromethane
DMTE	Dimethyltin bis(2-ethylhexyl mercaptoacetate)
EIC	Extracted Ion Chromatogram
ESI	Electrospray Ionization
HCD	Higher-Energy Collisional Dissociation
HMBC	Heteronuclear Multiple Bond Correlation
HPLC	High-Performance Liquid Chromatography
HRMS	High-Resolution Mass Spectrometry
HSQC	Heteronuclear Single Quantum Coherence
ICH	International Council for Harmonisation
IPA	Isopropanol
ISI	Internal Standard for Injection
LC	Liquid Chromatography
MDD	Multidimensional Decomposition
MS	Mass Spectrometry
NIAS	Non-Intentionally Added Substances
NMR	Nuclear Magnetic Resonance
NUS	Non-Uniform Sampling
NVP	N-Vinylpyrrolidin-2-one
OINDPs	Orally Inhaled and Nasal Drug Products
OSD	Oral Solid Dosage
PDPs	Parenteral Drug Products
PVC	Polyvinyl Chloride
PVP	Polyvinylpyrrolidone
PVPP	Polyvinyl Polypyrrolidone
RT	Retention Time
TMS	Tetramethylsilane
UHPLC	Ultra-High-Performance Liquid Chromatography
UPW	Ultrapure Water
UV	Ultraviolet
