# Supplementary material for: Leachable-Derived Impurities in Oral Solid Dosage Forms Generated by Solid-State Packaging–Excipient Interactions: A Case Study of a Polyvinyl Chloride Heat Stabilizer Reacting with Povidone
Source: Pharmaceutics. 2026 Sep 14;18(9):1153. doi: 10.3390/pharmaceutics18091153 (PMC13610799; doi:10.3390/pharmaceutics18091153)
Supplement: Supplementary file 1 [file pharmaceutics-18-01153-s001.zip › pharmaceutics-4505041-supplementary.pdf]

Supplementary Information for

Leachable-Derived Impurities in Oral Solid Dosage Forms Generated by Solid-State Packaging–Excipient Interactions: A Case Study of a Polyvinyl Chloride Heat Stabilizer Reacting with Povidone

E. Vaneeckhaute[1,2], J. Van Hooste[3], P. Weckx[1,2,], A. Teasdale[3], R. Cuyvers[3], J. Hammond[4], D. Wood[4], W. D'Autry[3], L. Martin[4],and E. Breynaert[1,2,]*

[1] NMRCoRe, NMR/X-Ray platform for Convergence Research, KU Leuven, Celestijnenlaan 200F - box 2461, B-3001 Leuven, Belgium.
[2] Center for Surface Chemistry and Catalysis – Characterisation and Application Team, KU Leuven, Celestijnenlaan 200F - box 2461, B-3001 Leuven, Belgium.
[3] Nelson Labs, Romeinsestraat 12, B-3001 Leuven, Belgium.
[4] Norgine Pharmaceutical Ltd, ARC Uxbridge, Building 01, Sanderson Road, Uxbridge, UB8 1DH, United Kingdom.
[*]Corresponding author: eric.breynaert@kuleuven.be

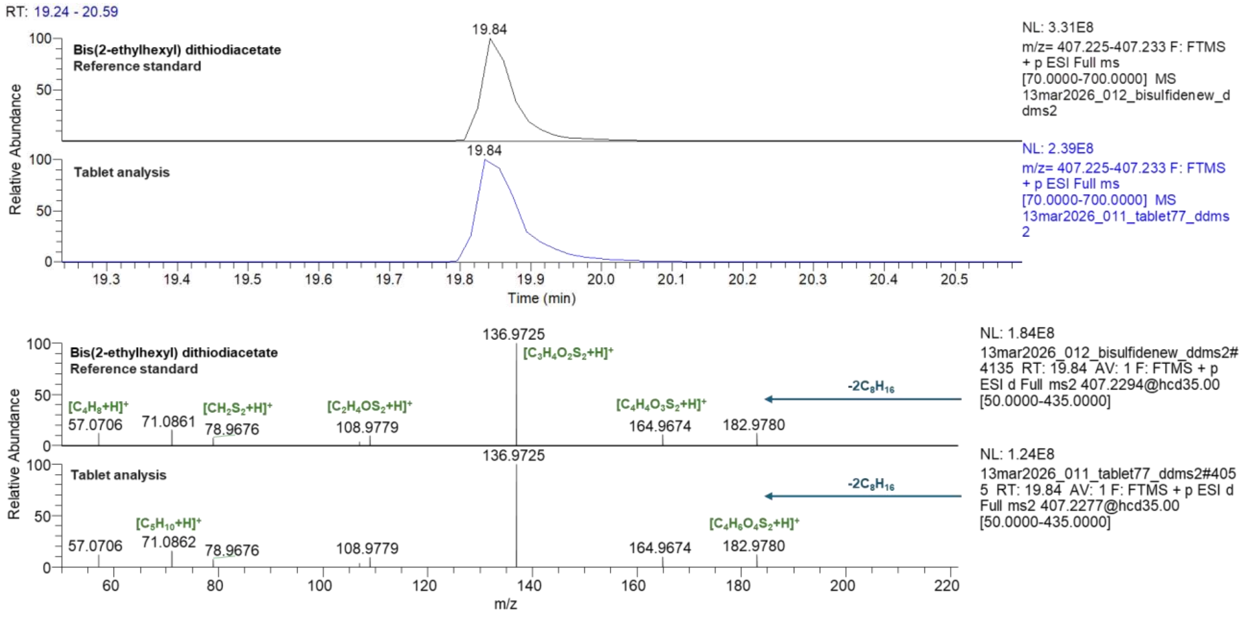

Figure S1: CHROMATOGRAMS: Top: Extracted Ion Chromatogram (EIC) of m/z +407.229 from the analysis of a reference standard of bis(2-ethylhexyl) dithiodiacetate (black); Bottom: EIC of m/z +407.2277 from the analysis of the drug product tablet (blue). MS SPECTRA: Top: MS/MS spectrum of precursor m/z +407.2 at 19.84 min from the analysis of a reference standard of bis(2-ethylhexyl) dithiodiacetate with relevant annotations of neutral losses (blue) and fragment ion formulas (green); Bottom: MS/MS spectrum of precursor m/z +407.2 at 19.84 min from the analysis of the drug product tablet with relevant annotations of neutral losses (blue) and fragment ion formulas (green).

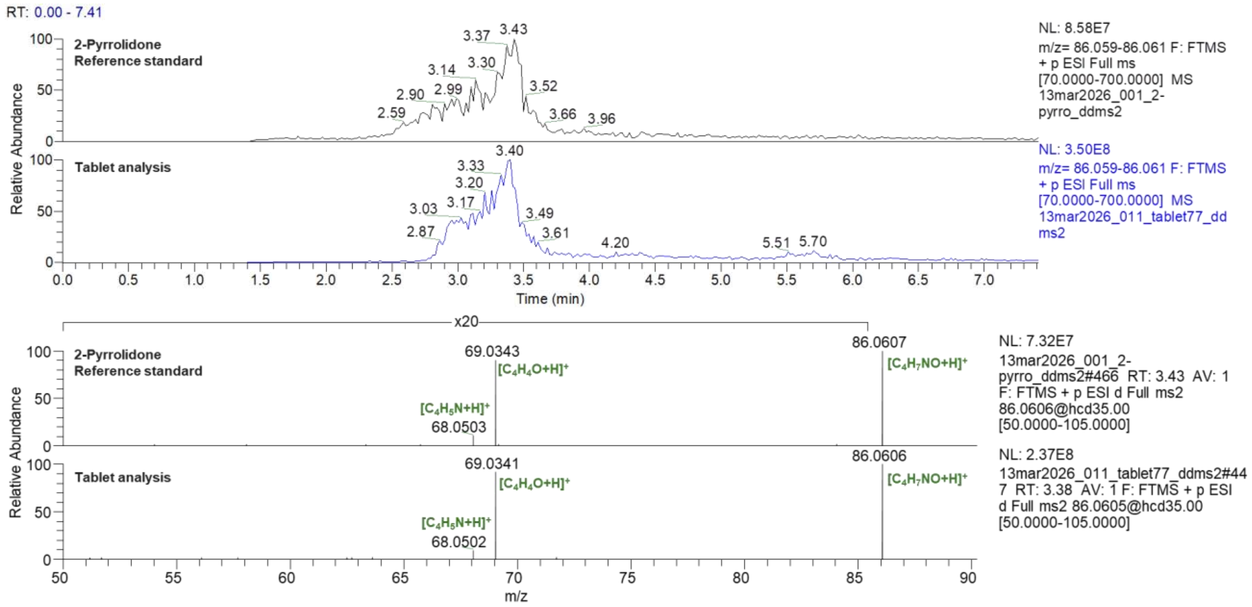

Figure S2: CHROMATOGRAMS: Top: Extracted Ion Chromatogram (EIC) of m/z +86.060 from the analysis of a reference standard of pyrrolidin-2-one (black); Bottom: EIC of m/z +86.060 from the analysis of the drug product tablet (blue). MS SPECTRA: Top: MS/MS spectrum of precursor m/z +86.1 at 3.43 min from the analysis of a reference standard of pyrrolidin-2-one with relevant annotations of fragment ion formulas (green); Bottom: MS/MS spectrum of precursor m/z +86.1 at 3.38 min from the analysis of the drug product tablet with relevant annotations of fragment ion formulas (green).

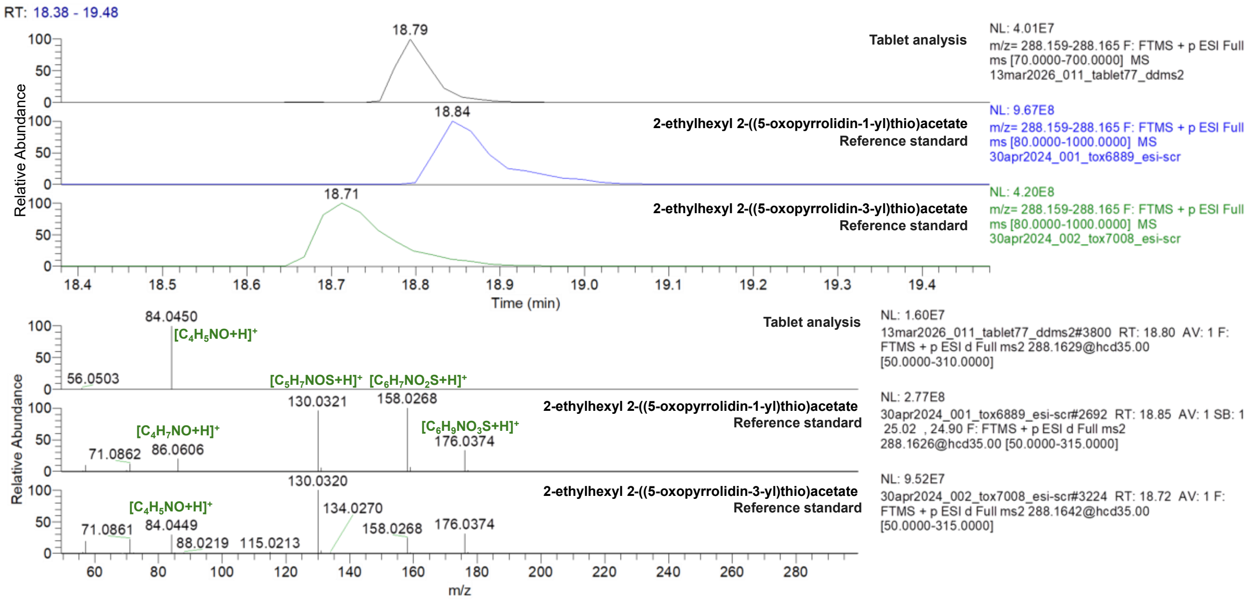

Figure S3: CHROMATOGRAMS: Top: Extracted Ion Chromatogram (EIC) of m/z +288.162 from the analysis of the drug product tablet (black); Middle: EIC of m/z +288.162 from the analysis of the synthesized reference standard of 2-ethylhexyl 2-((5-oxopyrrolidin-1-yl)thio)acetate (blue); Bottom: EIC of m/z +288.162 from the analysis of the synthesized reference standard of 2-ethylhexyl 2-((5-oxopyrrolidin-3-yl)thio)acetate (green). MS SPECTRA: Top: MS/MS spectrum of precursor m/z +288.2 at 18.80 min from the analysis of the drug product tablet with relevant annotations of fragment ion formulas (green); Middle: MS/MS spectrum of precursor m/z +288.2 at 18.85 min from the analysis of the synthesized reference standard of 2-ethylhexyl 2-((5-oxopyrrolidin-1-yl)thio)acetate with relevant annotations of fragment ion formulas (green); Bottom: MS/MS spectrum of precursor m/z +288.2 at 18.72 min from the analysis of the synthesized reference standard of 2-ethylhexyl 2-((5-oxopyrrolidin-3-yl)thio)acetate with relevant annotations of fragment ion formulas (green).

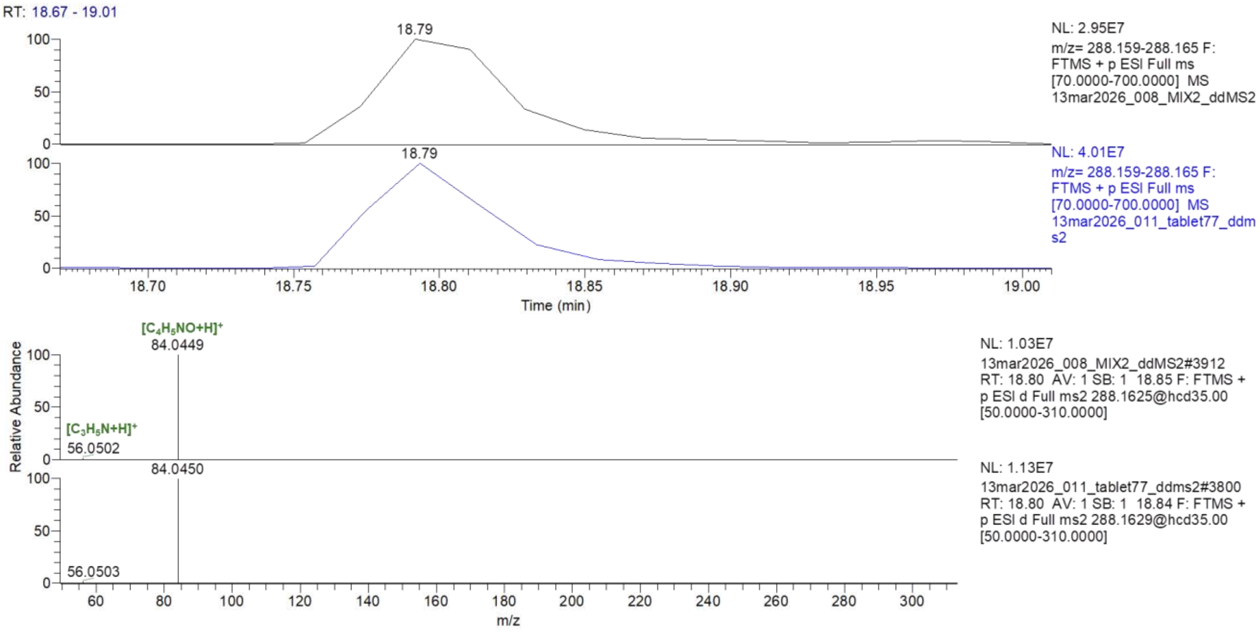

Figure S4: CHROMATOGRAMS: Top: Extracted Ion Chromatogram (EIC) of m/z +288.162 from the analysis of reaction mix 2 (pyrrolidin-2-one & DMTE) (black); Bottom: EIC of m/z +288.162 from the analysis of the drug product tablet (blue). MS SPECTRA: Top: MS/MS spectrum of precursor m/z +288.2 at 18.80 min from the analysis of reaction mix 2 (pyrrolidin-2-one & DMTE) with relevant annotations of fragment ion formulas (green); Bottom: MS/MS spectrum of precursor m/z +288.2 at 18.80 min from the analysis of the drug product tablet.

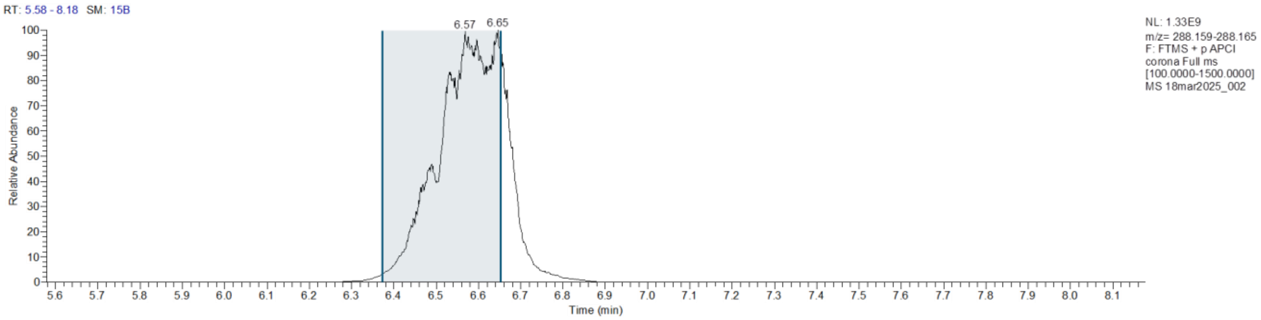

Figure S5: Extracted Ion Chromatogram (EIC) chromatogram of m/z +288.162 from the analysis of the synthesized impurity using the HPLC-UV/MS method adapted for the isolation of the target impurity of interest, as described in §3.2. The valve switch timing is indicated in blue, where the LC eluent is collected for impurity isolation. The timing is based on the MS retention time window of the target impurity of interest, taking into account a 0.10 min delay between the valve and MS detection.

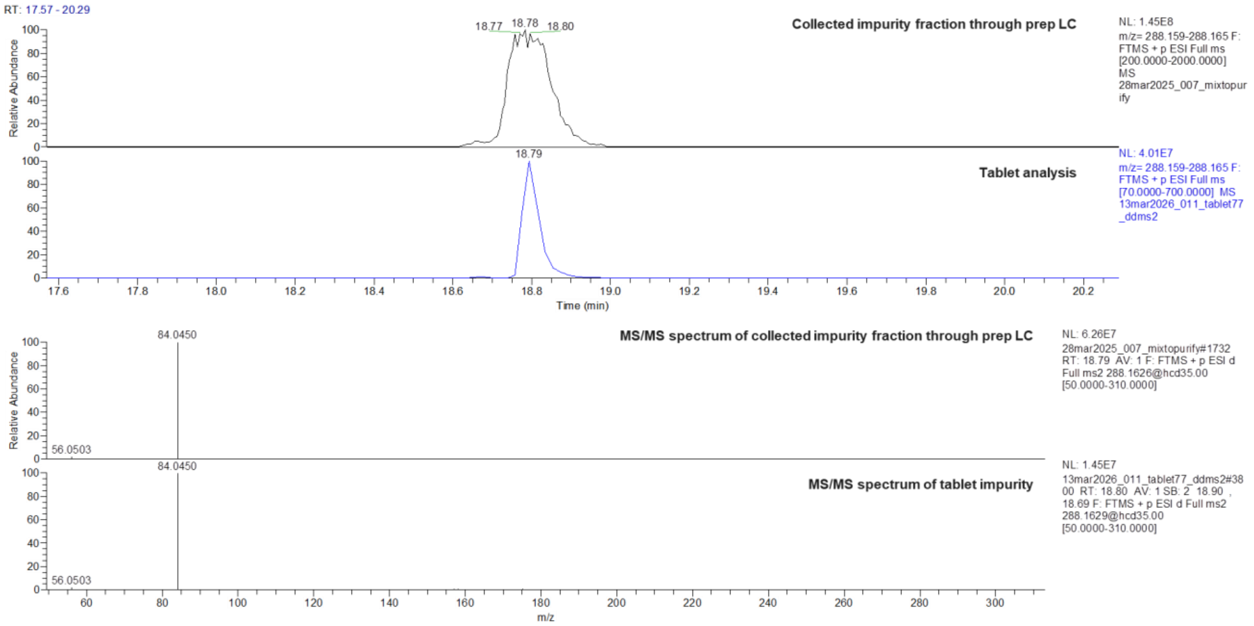

Figure S6: CHROMATOGRAMS: Top: Extracted Ion Chromatogram (EIC) of m/z +288.162 from the analysis of the collected fraction after preparative HPLC (black); Bottom: EIC of m/z +288.162 from the analysis of the drug product tablet (blue). MS SPECTRA: Top: MS/MS spectrum of precursor m/z +288.2 at 18.79 min from the collected fraction after preparative HPLC; Bottom: MS/MS spectrum of precursor m/z +288.2 at 18.80 min from the analysis of the drug product tablet.

Table S1. Overview of the ^{1}H and ^{13}C NMR signals observed for 140 mM 2-ethylhexyl 2-((5-oxopyrrolidin-2-yl)thio)acetate referenced towards tetramethylsilane (TMS) in deuterated chloroform ($CDCl_3$). *The methine at position 5” in the pyrrolidone ring was taken as the integration reference of unity. **Due to chemical shift overlap of the 6’ and 8’ end terminal methyls, the integration was divided by two to represent the relative ration to the reference.

Position	Description	^{1}H (ppm)	Integral	^{13}C (ppm)
Pyrrolidinone ring				
1” NH	tertiary amine	6.929	0.98	/
2” C=O	lactam carbonyl	/	/	177.164
3”	methylene diastereotopic	2.487/2.338	2.13	29.310
4”	methylene diastereotopic	2.590/2.071	2.1	28.446
5”	methine to sulfur	4.964	1*	60.420
Thioacetate linker				
1 C=O	ester carbonyl	/	/	171.070
2	methylene to sulfur	3.341	2.05	33.224
Main alkyl chain				
1’	methylene to oxygen	4.067	2.17	68.315
2’	methine branch	1.605	1.13	38.687
3’	methylene	1.301	6.7	30.266
4’	methylene	1.286		28.867
5’	methylene	1.296		22.966
6’	methyl terminal	0.899	3.2**	10.950
Branch alkyl chain				
7’	methylene	1.367	2.25	23.648
8’	methyl terminal	0.900	3.2**	14.066
Solvents				
$CDCl_3$	chloroform	7.274	/	77.245
Reference				
TMS	tetramethylsilane	0.000	/	0.000

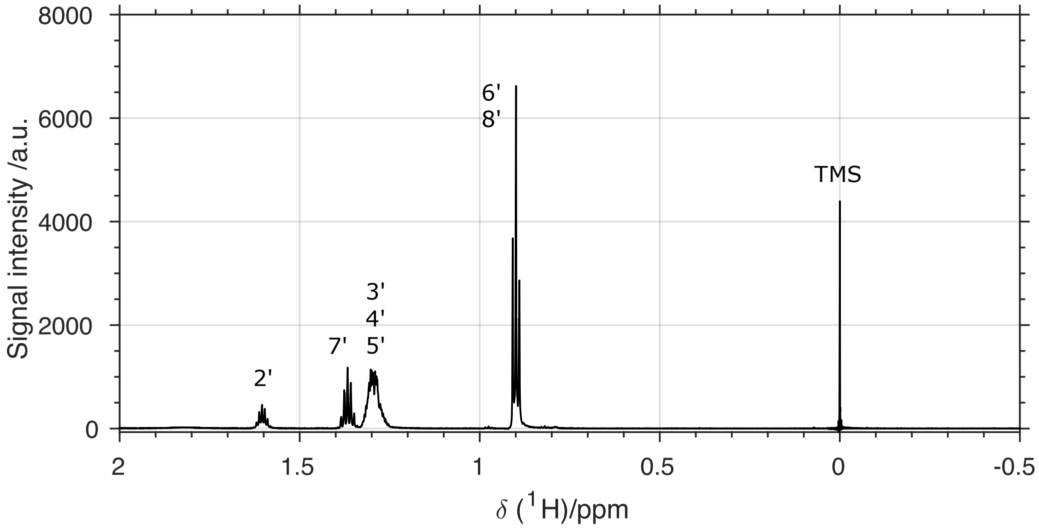

Figure S7. Detail of ^{1}H NMR spectrum from -0.5 to 2 ppm of the purified impurity (801 MHz, 298K, $CDCl_3$).

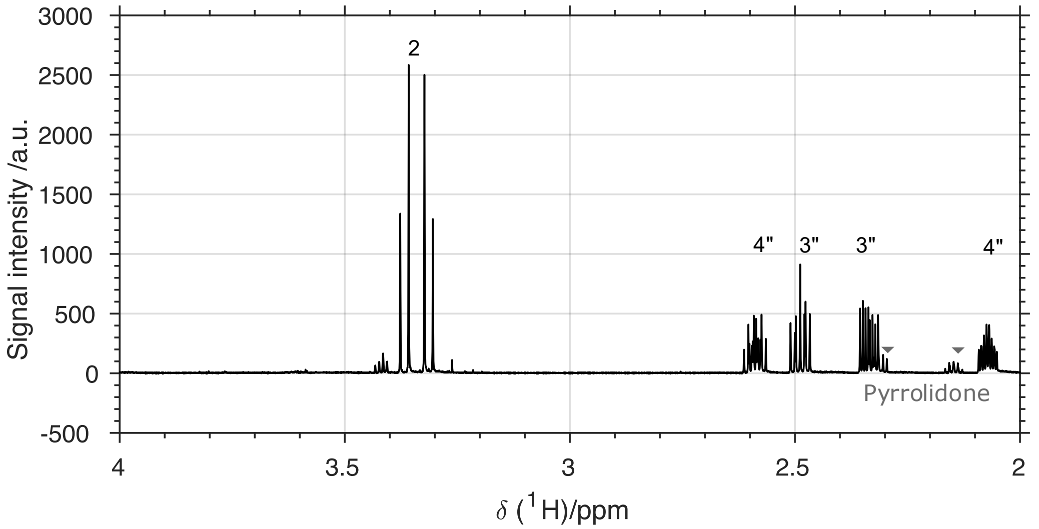

Figure S8. Detail of ^{1}H NMR spectrum from 2 to 4 ppm of the purified impurity (801 MHz, 298K, $CDCl_3$).

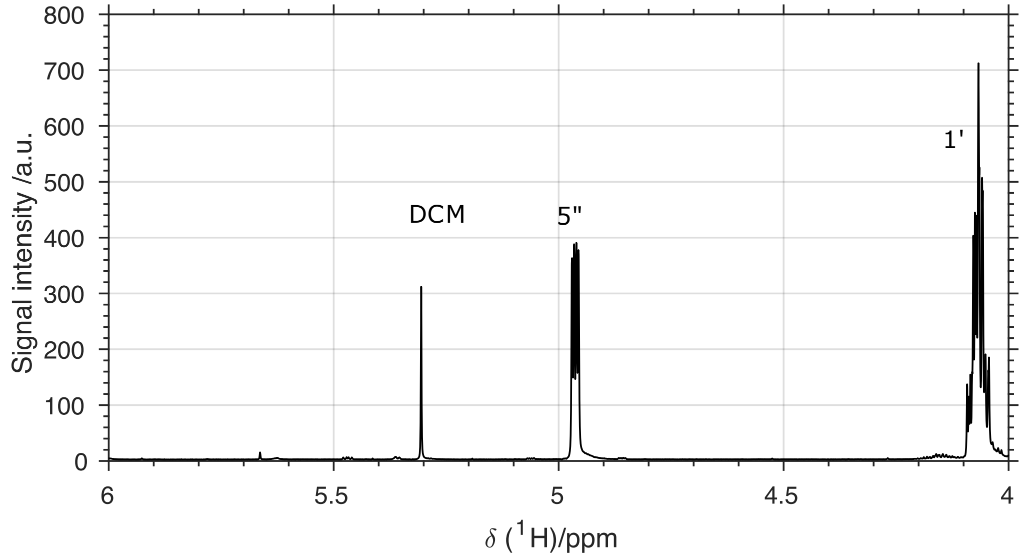

Figure S9. Detail of ^{1}H NMR spectrum from 4 to 6 ppm of the purified impurity (801 MHz, 298K, $CDCl_3$). Dichloromethane (DCM) was found as an impurity common in $CDCl_3$.

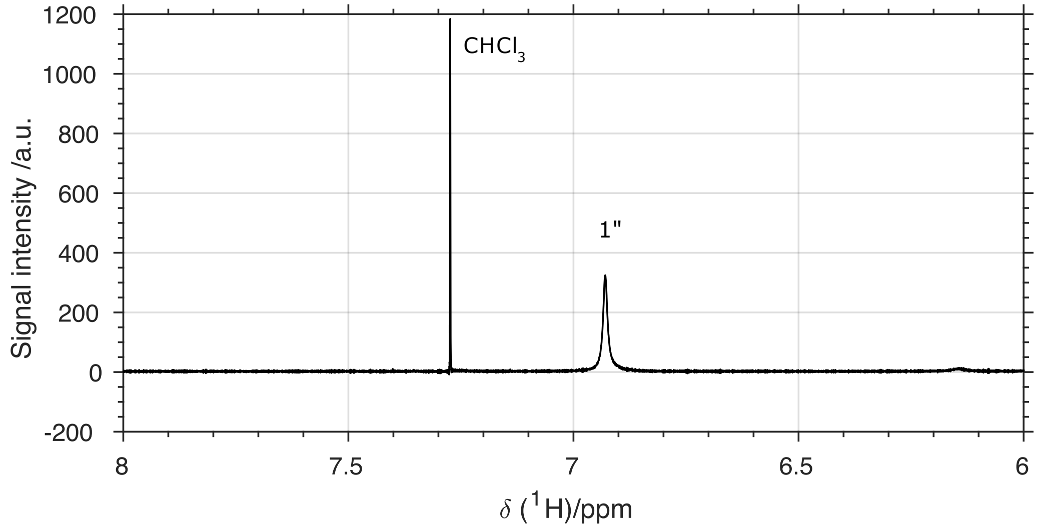

Figure S10. Detail of ^{1}H NMR spectrum from 6 to 8 ppm of the purified impurity (801 MHz, 298K, $CDCl_3$). Trace protonated chloroform is shown here.

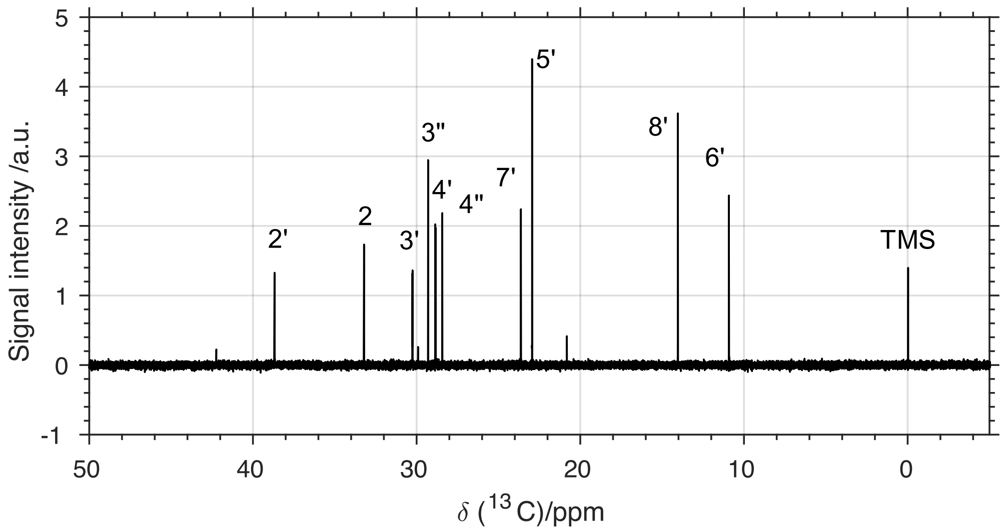

Figure S11. Detail of ^{13}C NMR spectrum from -5 to 50 ppm of the purified impurity (201 MHz, 298K, $CDCl_3$).

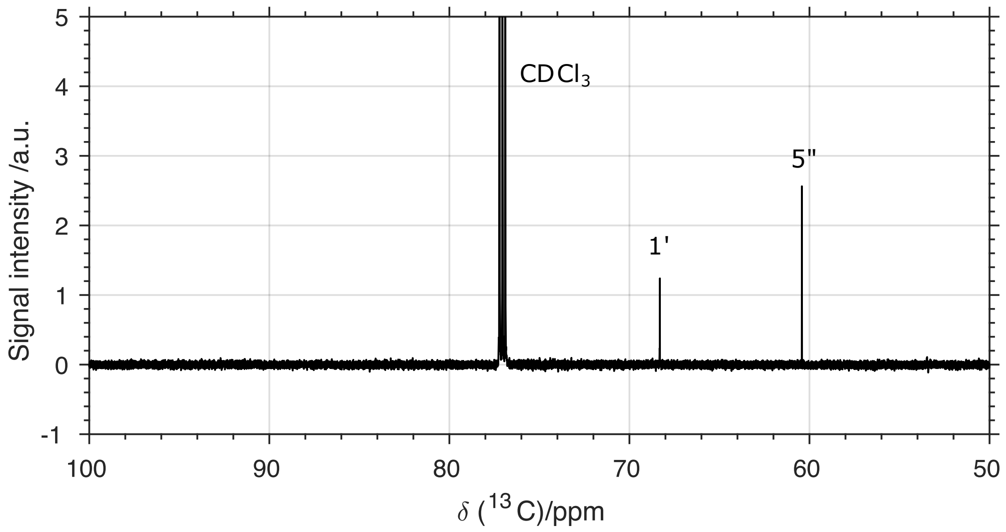

Figure S12. Detail of ^{13}C NMR spectrum from 50 to 100 ppm of the purified impurity (201 MHz, 298K, $CDCl_3$).

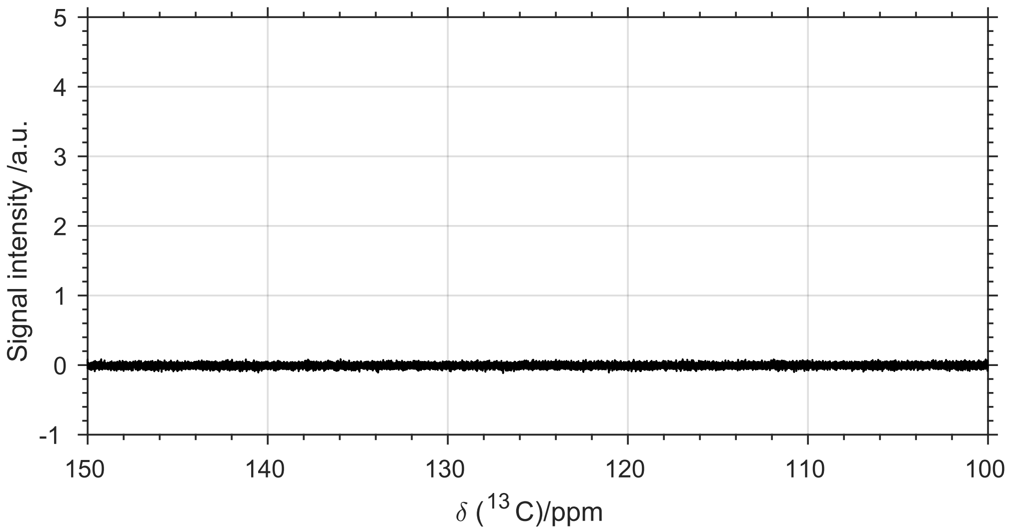

Figure S13. Detail of ^{13}C NMR spectrum from 100 to 150 ppm of the purified impurity (201 MHz, 298K, $CDCl_3$).

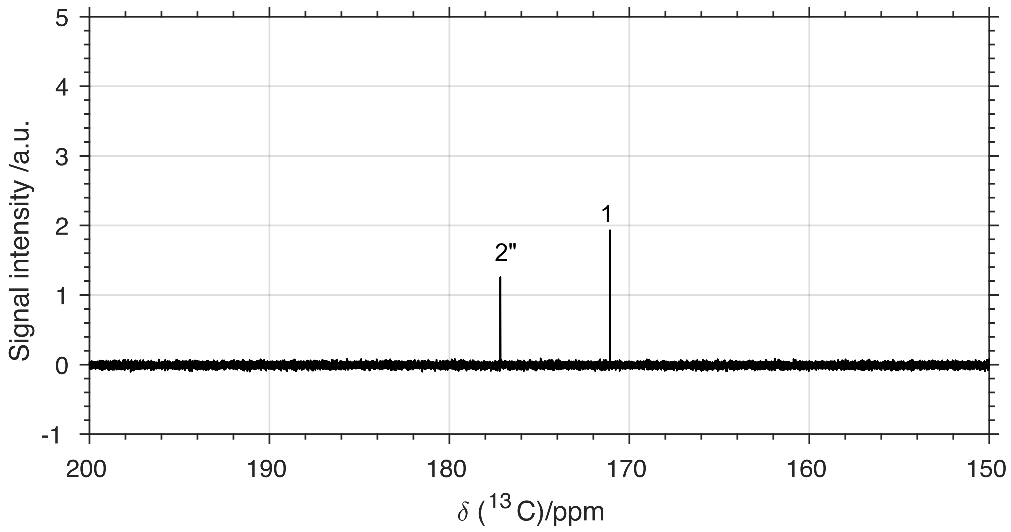

Figure S14. Detail of ^{13}C NMR spectrum from 150 to 200 ppm of the purified impurity (201 MHz, 298K, $CDCl_3$).

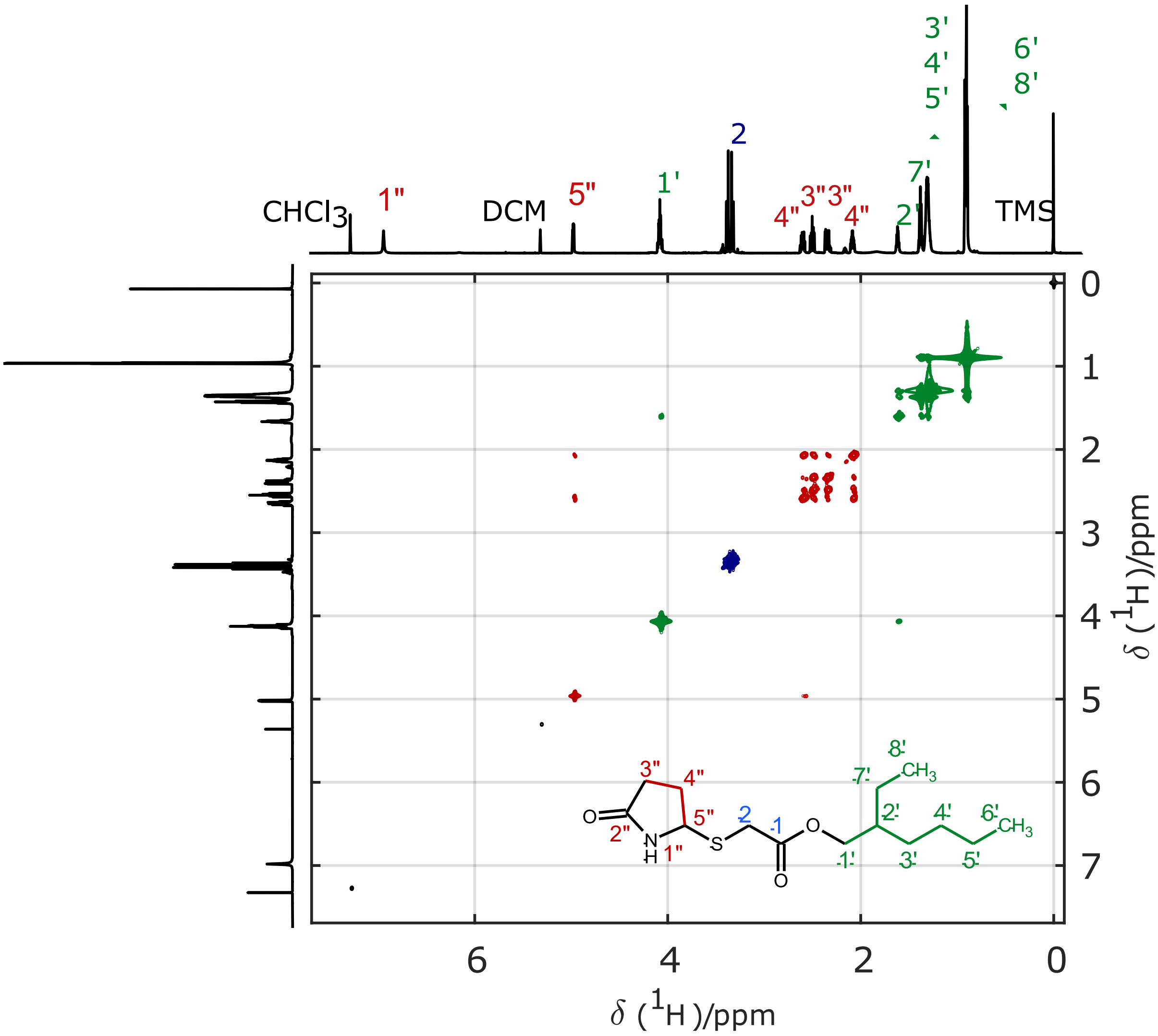

Figure S15. ^1H–^1H COSY spectrum of the purified impurity (801 MHz, 298K, $CDCl_3$) with 2048 points in F2 and 256 points in F1 with 25% NUS.

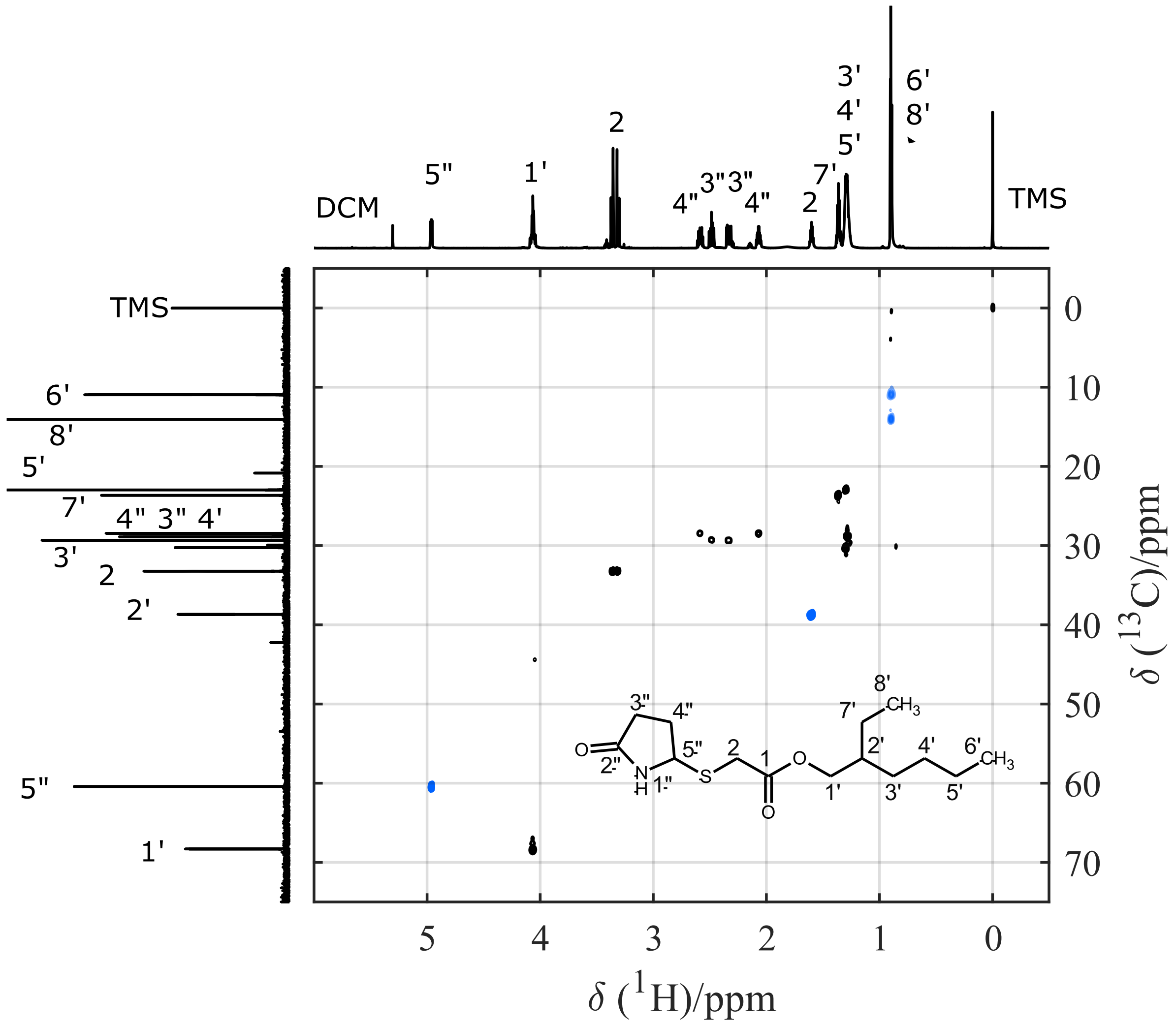

Figure S16. ^{1}H–^{13}C HSQC spectrum of the purified impurity (801 MHz and 201 MHz, 298K, $CDCl_3$) with 2048 points in F2 and 256 points in F1 with 25% NUS. Blue correlations are CH/CH_3 multiplicity-edited. Black correlations are CH_2 multiplicity-edited.

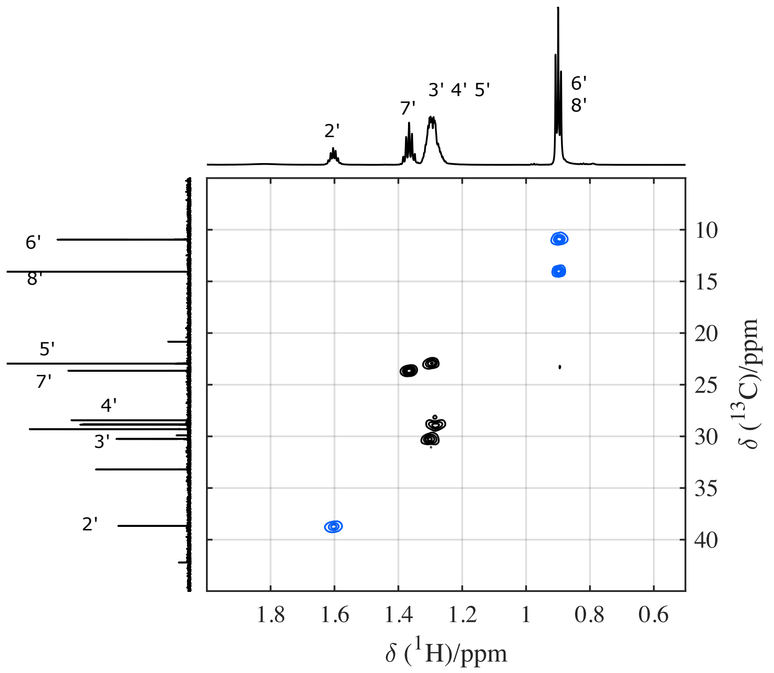

Figure S17. Detail of ^{1}H–^{13}C HSQC spectrum from 0.5 to 2 ppm in F2 and 5 to 45 ppm in F1 of the purified impurity (801 MHz and 201 MHz, 298K, $CDCl_3$). Blue correlations are CH/CH3 multiplicity-edited. Black correlations are CH_2 multiplicity-edited.

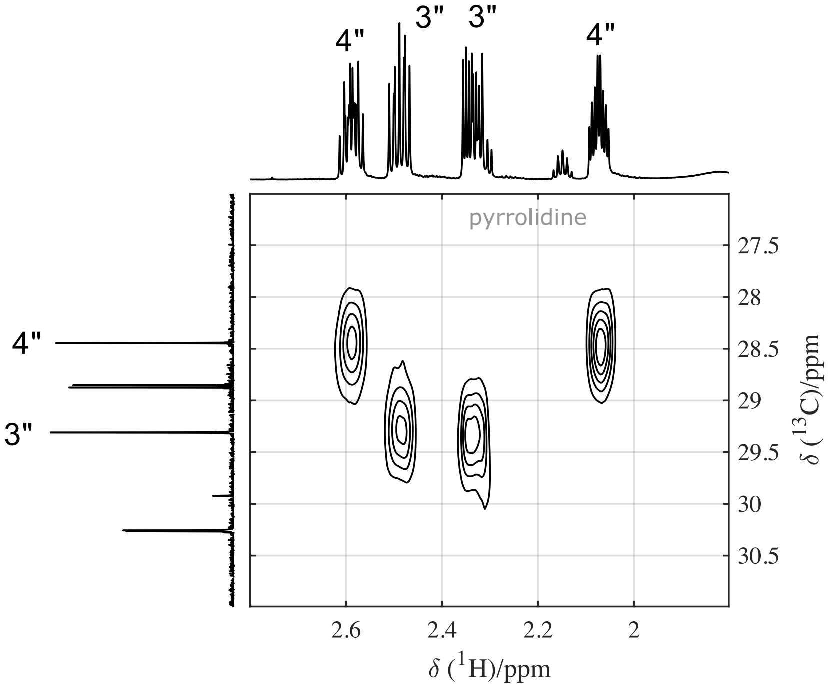

Figure S18. Detail of ^{1}H–^{13}C HSQC spectrum from 1.8 to 2.8 ppm in F2 and 27 to 31 ppm in F1 of the purified impurity (801 MHz and 201 MHz, 298K, $CDCl_3$). Black correlations are CH_2 multiplicity-edited.

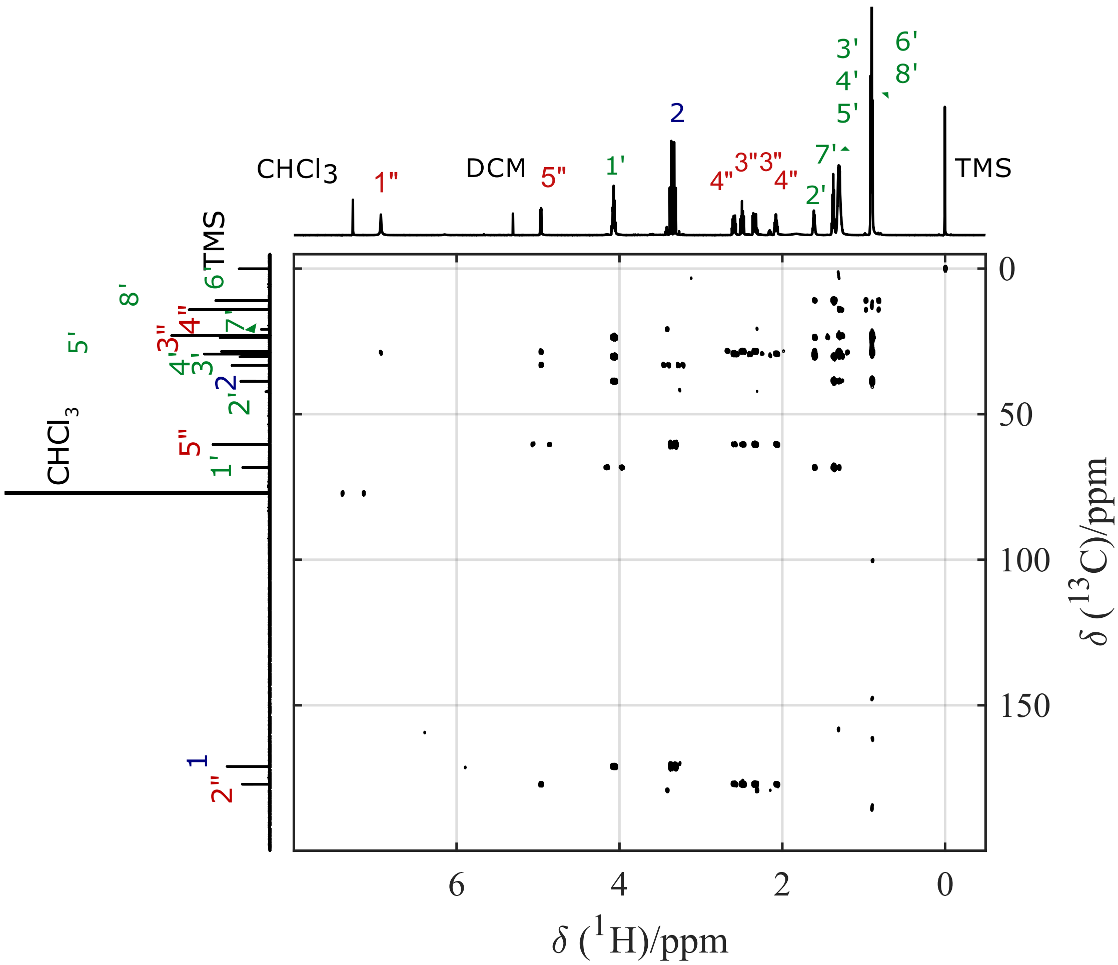

Figure S19. ^{1}H–^{13}C HMBC spectrum of the purified impurity (801 MHz and 201 MHz, 298K, $CDCl_3$) with 4096 points in F2 and 256 points in F1 with 25% NUS. A detailed plot is shown in Figure S20.

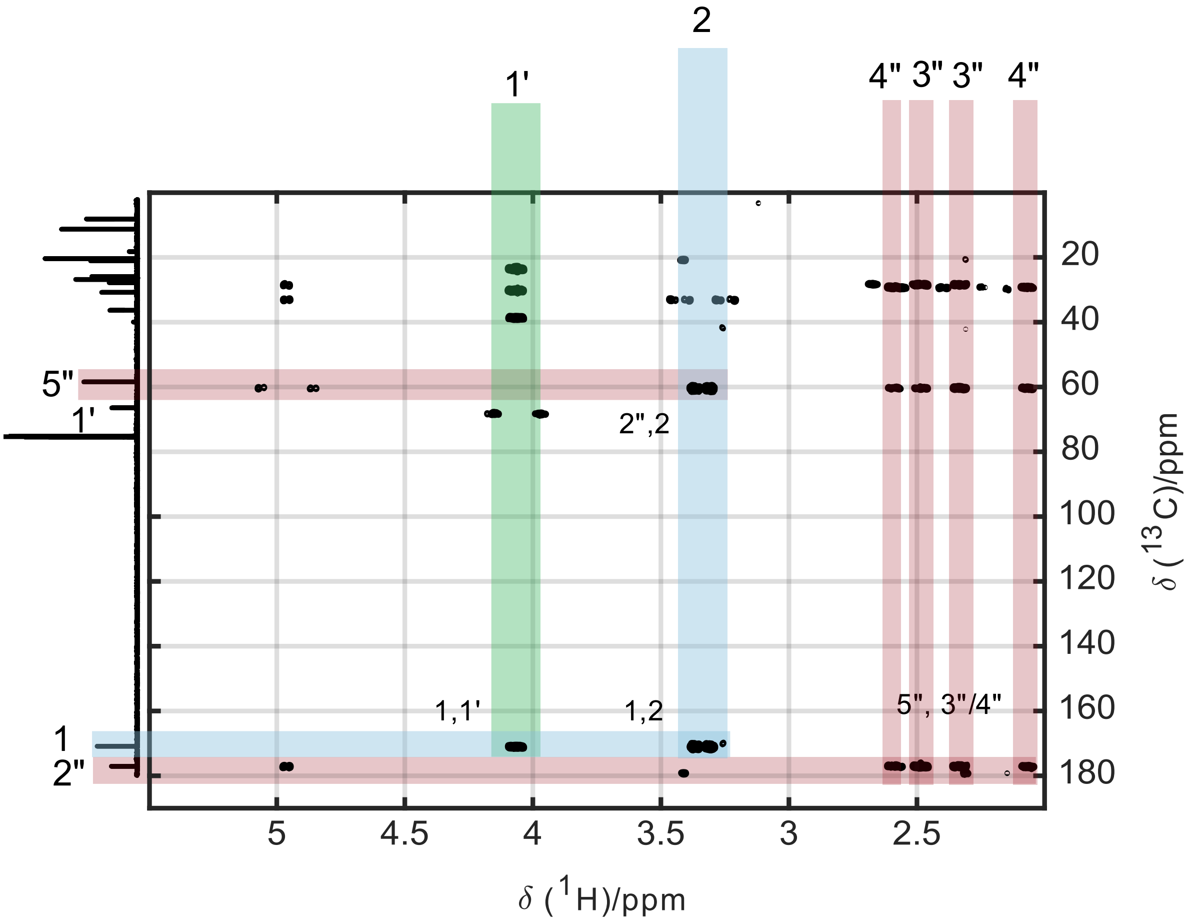

Figure S20. Detail ^{1}H–^{13}C HMBC spectrum from 2 ppm to 5.5 ppm of the purified impurity (801 MHz and 201 MHz, 298K, $CDCl_3$) with 4096 points in F2 and 256 points in F1 with 25% NUS.

Performing an experiment where equimolar amounts of pyrrolidine-2-one & bis(2-ethylhexyl) dithiodiacetate OR pyrrolidine-2-one & DMTE are mixed under the exact same conditions, the yield of the target impurity of interest is approximately 1 order of magnitude higher in the reaction mix with DMTE, as shown in the comparative chromatograms below.

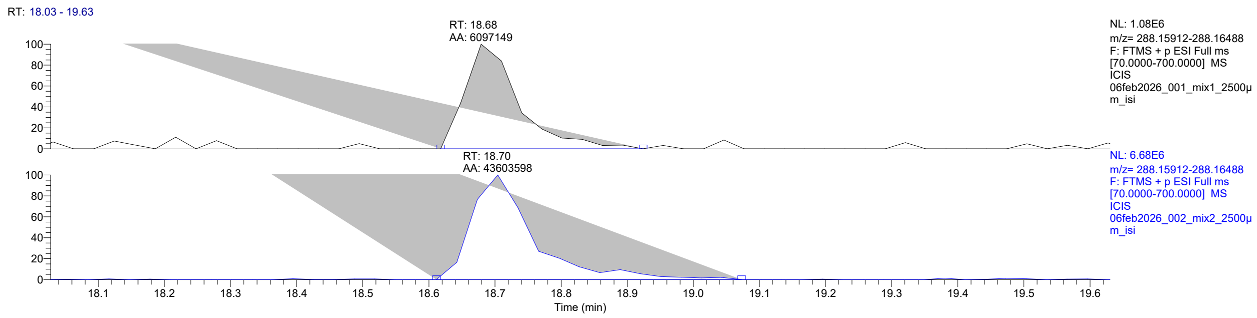

Figure S21: Top: Extracted Ion Chromatogram (EIC) of m/z +288.161 from the analysis of reaction mix 1 (equimolar 2500 µM amounts of pyrrolidine-2-one & bis(2-ethylhexyl) dithiodiacetate) (black). Bottom: EIC of m/z +288.161 from the analysis of reaction mix 2 (equimolar 2500 µM amounts of pyrrolidine-2-one & DMTE) (blue).

IPA extraction data of the several blister packaging components indicate that DMTE clearly is extracted from the PVC base foil and is not detected in the aluminum top foil. The printing does not show any significant difference in the DMTE concentration levels when analyzing a printed or a non-printed blister packaging. The data are shown in the LC/MS data of the several IPA extracts, showing extracted ion chromatograms specific for the DMTE compound:

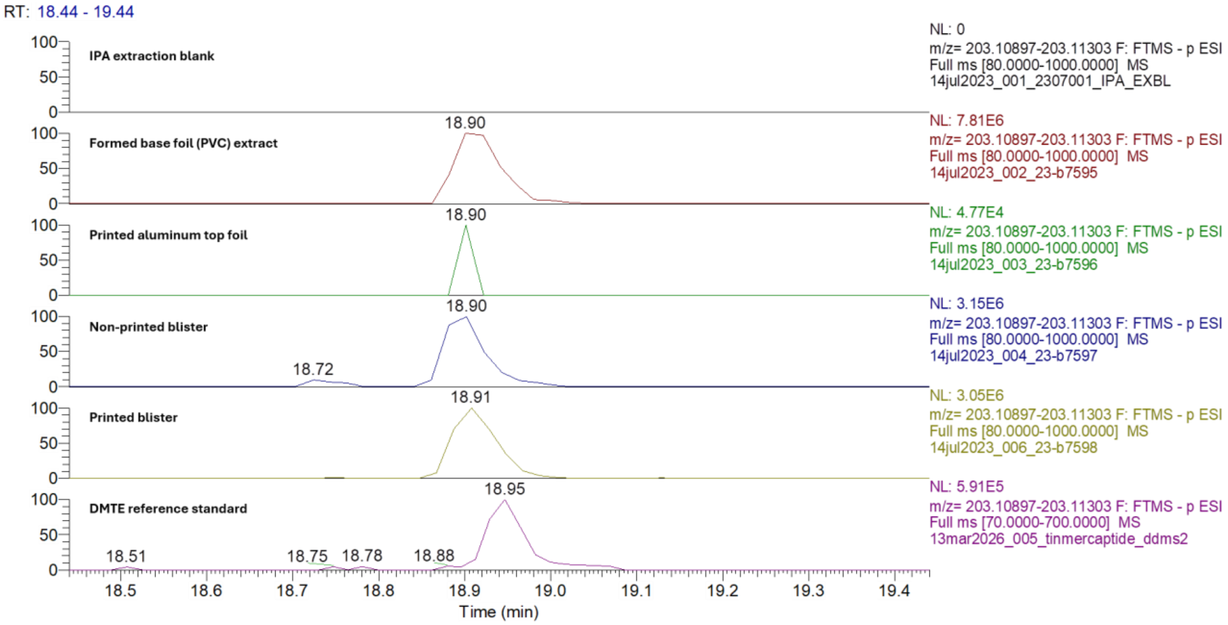

Figure S22: LC/MS data of several blister packaging components after closed vessel incubation extraction (submersion) in isopropanol (IPA). Extraction Ion Chromatograms (EIC) of m/z -203.111, the diagnostic ion for DMTE, from top to bottom:

- The IPA extraction blank
- The PVC base foil
- The printed aluminum top foil
- The non-printed blister (PVC base + aluminum top foil)
- The printed blister (PVC base + aluminum top foil)
- An authentic reference standard of DMTE (the small retention time difference can be explained by the fact that this analysis has not run in the same sequence)
